# Transcription cofactor GRIP1 differentially affects myeloid cell–driven neuroinflammation and response to IFN-β therapy

**DOI:** 10.1084/jem.20192386

**Published:** 2020-10-12

**Authors:** Sanda Mimouna, David A. Rollins, Gayathri Shibu, Bowranigan Tharmalingam, Dinesh K. Deochand, Xi Chen, David Oliver, Yurii Chinenov, Inez Rogatsky

**Affiliations:** 1 The David Z. Rosensweig Genomics Center, Hospital for Special Surgery Research Institute, New York, NY; 2 Graduate Program in Immunology and Microbial Pathogenesis, Weill Cornell Graduate School of Medical Sciences, New York, NY

## Abstract

Macrophages (MФ) and microglia (MG) are critical in the pathogenesis of multiple sclerosis (MS) and its mouse model, experimental autoimmune encephalomyelitis (EAE). Glucocorticoids (GCs) and interferon β (IFN-β) are frontline treatments for MS, and disrupting each pathway in mice aggravates EAE. Glucocorticoid receptor–interacting protein 1 (GRIP1) facilitates both GR and type I IFN transcriptional actions; hence, we evaluated the role of GRIP1 in neuroinflammation. Surprisingly, myeloid cell–specific loss of GRIP1 dramatically reduced EAE severity, immune cell infiltration of the CNS, and MG activation and demyelination specifically during the neuroinflammatory phase of the disease, yet also blunted therapeutic properties of IFN-β. MФ/MG transcriptome analyses at the bulk and single-cell levels revealed that GRIP1 deletion attenuated nuclear receptor, inflammatory and, interestingly, type I IFN pathways and promoted the persistence of a homeostatic MG signature. Together, these results uncover the multifaceted function of type I IFN in MS/EAE pathogenesis and therapy, and an unexpectedly permissive role of myeloid cell GRIP1 in neuroinflammation.

## Teaser

Mimouna et al. use functional and transcriptomic approaches in a mouse model of multiple sclerosis to show that a transcriptional coregulator, GRIP1, known for anti-inflammatory actions, promotes macrophage- and microglia-driven neuroinflammation but also mediates therapeutic efficacy of type I IFN.

## Graphical Abstract

**Figure:**
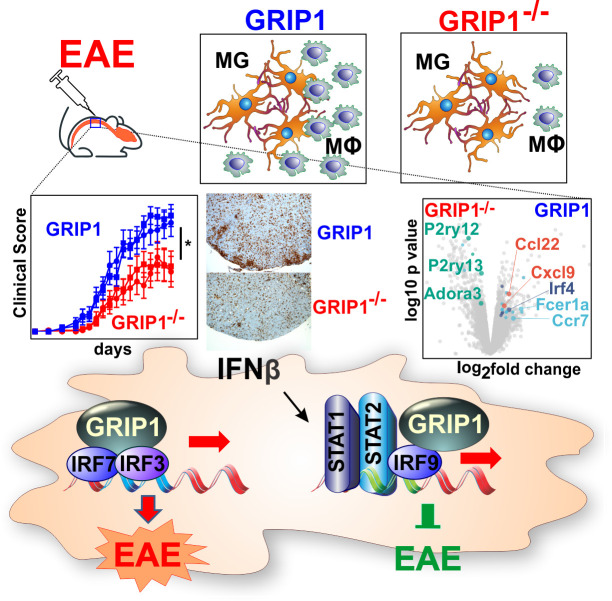

## Introduction

Multiple sclerosis (MS) is a chronic inflammatory disease that affects the central nervous system (CNS) and whose etiology remains unknown (Bishop and Rumrill, 2015; Dendrou et al., 2015; Lassmann, 2011). Clinically, four types of MS have been described: primary progressive MS; secondary progressive MS; progressive relapsing; and, the most common, relapsing-remitting MS (RRMS; Milo and Miller, 2014). For all types, autoimmune demyelination is the hallmark of the disease, which prompted much work dissecting the roles of T cells (Jäger et al., 2009; Kaskow and Baecher-Allan, 2018; Liu et al., 2008; McGinley et al., 2018; Merrill et al., 1992) and B cells (Negron et al., 2019; Staun-Ram and Miller, 2017; Weber et al., 2010) in MS. However, recent accumulating evidence demonstrates the pivotal role of myeloid cells such as microglia (MG) in MS pathogenesis (Croxford et al., 2015; Mahad and Ransohoff, 2003; Mishra and Yong, 2016; Sominsky et al., 2018; Yamasaki, 2014). MG are CNS-resident specialized macrophage (MФ)-like cells with a ramified morphology and motile processes that enable MG to migrate throughout the CNS, constantly surveying the environment and responding accordingly if any change is detected. In healthy conditions, they ensure brain homeostasis by pruning neurons, clearing debris, and providing neurotrophic factors during development and adult life (Hagemeyer et al., 2017; Kierdorf and Prinz, 2017). MG and MФ share a common erythromyeloid progenitor, but they part ways very early in development (embryonic day 9.5 [E9.5]), when MG migrate into the fetal brain, where they maintain their pool through self-renewal (Ginhoux et al., 2010; Kierdorf et al., 2013). In contrast, MФ rely on bone marrow (BM)–derived precursors for renewal and are able to circulate into the blood as monocytes or reside in tissues, depending on their role and immunological state (Goldmann et al., 2016). Both cell types display high plasticity (Holtman et al., 2017; Italiani and Boraschi, 2014; Murray, 2017; Shemer et al., 2015) and can have similar roles, especially during inflammation. In disease, such as MS, together with CNS-infiltrating MФ, MG shape the immune responses through antigen presentation, phagocytosis of myelin, and cytokine secretion (Almolda et al., 2011; Fourgeaud et al., 2016; Franco and Fernández-Suárez, 2015). These functions place MG and MФ as central effectors of neuroinflammation, but their specific and potentially divergent contribution to MS pathogenesis remains poorly defined.

Recent genomic and transcriptomic tools made it possible to better characterize the myeloid cells of the CNS, and especially MG, by building the “microgliome” (Gosselin et al., 2017; Holtman et al., 2017; Sousa et al., 2017). An increasing number of studies are investigating the transcriptional signatures of MG and MФ at homeostasis and during MS or experimental autoimmune encephalomyelitis (EAE), a commonly used mouse model for RRMS (Holtman et al., 2017; Sevastou et al., 2016; van der Poel et al., 2019). These studies showed that, apart from the surface proteins shared by these two cell types (e.g., Cd45, Cd11b), certain markers are MG specific (Tmem119/Sall1) or MФ specific (Ccr2), illustrating not only distinct ontology of these cells but also their different responses depending on the local environment (Bennett et al., 2016; Buttgereit et al., 2016; Gu et al., 2016; Koeniger and Kuerten, 2017). Nevertheless, during neuroinflammation, MФ infiltrate the CNS together with the bulk of immune cells and, along with MG, become activated, which shifts the transcriptomic makeup and, consequently the repertoire of molecules expressed on their surface, making these cells harder to distinguish from each other (Greter et al., 2015; Prinz et al., 2011).

There is no cure for MS; however, glucocorticoid (GC) hormones and type I IFN (specifically, IFN-β) are used to alleviate MS symptoms (Goodin, 2014; Vosoughi and Freedman, 2010; Wingerchuk and Carter, 2014). GC hormones are potent anti-inflammatory drugs that are also essential for preventing irreversible neuronal damage during MS flares (Goodin, 2014; Smets et al., 2017). They act through the GC receptor (GR), a ligand-dependent transcription factor that localizes to specific genomic binding sites and activates anti-inflammatory genes (e.g., *Dusp1*, *Tsc22d3*) or, by tethering to nonreceptor transcription factors AP1 and NF-κB, represses proinflammatory ones (e.g., *Tnf*, *Il1b*; Nissen and Yamamoto, 2000; Sacta et al., 2018; Uhlenhaut et al., 2013). Interestingly, a unique p160/NCoA GR coregulator—GR–interacting protein 1 (GRIP1/NCoA2/TIF2)—facilitates both GR-mediated activation and repression (Chinenov et al., 2012; Lee et al., 2002; Rollins et al., 2017). In fact, loss of GRIP1 in myeloid cells such as MФ leads to a dramatic derepression of numerous inflammatory mediators, which in vivo sensitizes mice to acute LPS-induced sepsis and chronic high-fat diet–induced metabolic inflammation (Chinenov et al., 2012; Coppo et al., 2016; Rollins et al., 2017).

IFN-β is prescribed to RRMS patients to delay relapses and disease progression (Bermel and Rudick, 2007). The type I IFN pathway is triggered upon TLR3 activation that, through a series of adapter proteins, leads to activating phosphorylation of IFN-regulatory factors 3/7 (IRF3/7) that bind IFN-stimulated response elements and initiate the IFN-β gene transcription. Newly produced IFN-β acts in a para- and autocrine manner via the IFN-α/β receptor (IFNAR) at the cell surface, inducing the second wave of signaling through JAK/STAT phosphorylation and assembly of the ISGF3 (STAT1/STAT2/IRF9) transcription complex that binds IFN-stimulated response elements and activates numerous IFN-stimulated genes (ISGs; Chen et al., 2017). Studies in mice showed that a whole-body KO of IFN-β worsens EAE (Teige et al., 2003) and that conditional IFNAR-KO in myeloid cells or GR-KO in hematopoietic cells also leads to more severe disease and enhanced lethality, lending genetic support to therapeutic efficacy of IFN-β in EAE and MS (Prinz et al., 2008; Wüst et al., 2008). The protective role of IFN-β in MS, however, is puzzling in light of the well-established pathogenic role of type I IFN in other autoimmune diseases, such as systemic lupus erythematosus, Sjögren syndrome, and neuromyelitis optica, to name a few (Axtell et al., 2011; Crow, 2014). The exact mechanisms underlying a beneficial function of IFN-β in MS remain obscure. Unexpectedly, we discovered that GRIP1 physically interacts with several members of the IRF family and potentiates type I IFN signaling in MФ in conjunction with IRFs 3, 7, and 9 (Flammer et al., 2010; Reily et al., 2006). The contribution of GRIP1 to the type I IFN network in vivo has never been assessed.

Given that MФ GRIP1 cooperated with both GRs and IRFs, transcription factors that reportedly mediate neuroprotection in MS, we sought to assess the function of this coregulator during neuroinflammation. Here, using mice conditionally lacking GRIP1 in myeloid cells, we describe an unexpected impact of GRIP1 on the neuroinflammatory phase of EAE, potentially pointing to different roles it plays in MG versus peripheral MФ. We analyze transcriptomic changes that occur in the myeloid compartment of the CNS at homeostasis and during EAE at the bulk and single-cell levels. Finally, we present data on GRIP1 driving the effect of a frontline treatment of MS in mice with EAE.

## Results

### GRIP1 regulates the inflammatory transcriptome in P0 MG in vitro

**Figure S1.**
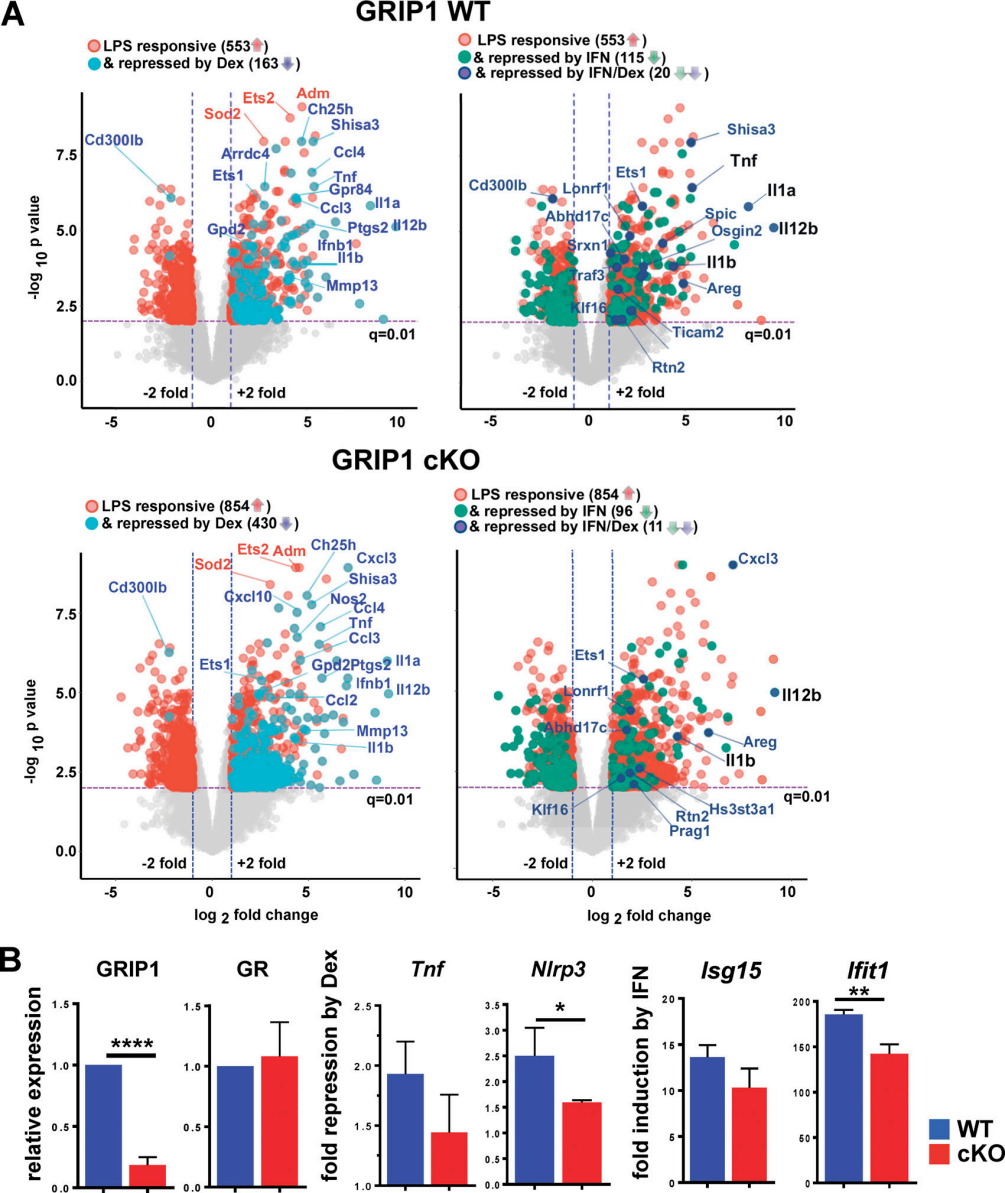
**Characterization of P0 MG gene expression in vitro. (A)** Gene expression in MG derived in vitro from WT and cKO P0 pups (see Materials and methods) and treated for 2 h with 10 ng/ml LPS ± 100 nM Dex or 10 ng/ml LPS ± 500 U/ml IFN-β was profiled by RNAseq (*n* = 2 with multiple neonates pooled for each experiment). Volcano plots (fold change = 2; FDR P < 0.05) show genes regulated by LPS (red) in WT (963 total; 553 upregulated) and cKO (1,403 total; 854 upregulated) overlaid with genes downregulated by Dex (teal; 163 and 460 in WT and cKO, respectively), IFN-β (green; 115 and 96 in WT and cKO, respectively), or both (dark blue; 20 and 11 in WT and cKO, respectively). Shown in black are key inflammatory cytokines upregulated by LPS and downregulated by Dex as well as IFN-β. **(B)** Neonatal WT and cKO MG were treated for 2 h with LPS ± Dex or with IFN-β, and expression of indicated genes was assessed by RT-qPCR with *Actb* used for normalization. Relative expression of GRIP1 and GR mRNA in the cKO are shown relative to that in WT (= 1; *n* = 4). Fold repression by Dex = [RNA]_LPS_/[RNA]_LPS+Dex_ (*n* = 5). Induction of ISGs by IFN-β is shown relative to untreated (= 1; *n* = 4). Shown are mean ± SD; two-tailed Student’s *t* test; *, P < 0.05; **, P < 0.01; ****, P < 0.0005.

**Figure 1.**
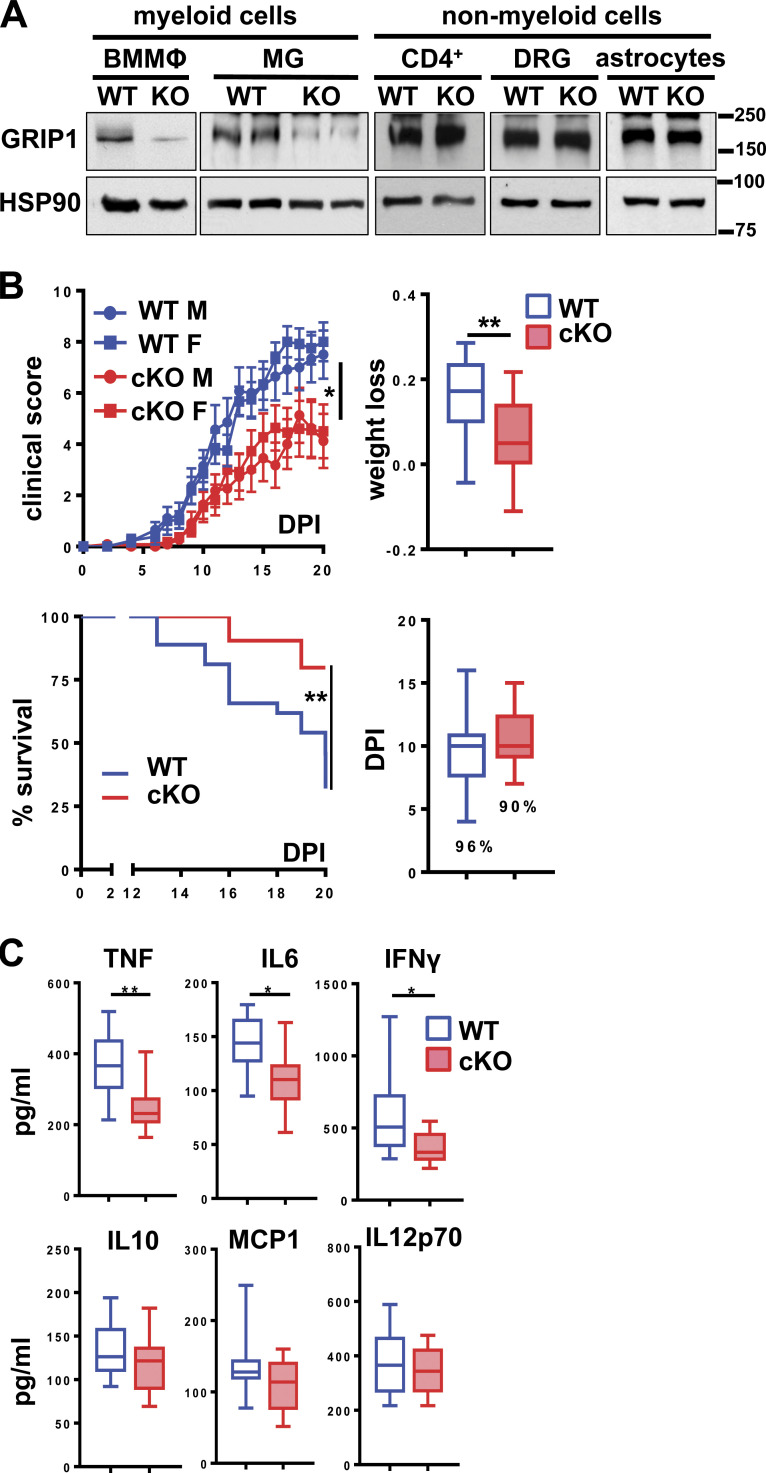
**Myeloid cell–specific GRIP1 deletion ameliorates EAE. (A)** GRIP1 Western blots of BMMФ, MG expanded in vitro from P0 pups, spleen CD4^+^ T cells, dorsal root ganglia (DRG) neurons, and astrocytes from WT and GRIP1-cKO mice; heat shock protein 90 (HSP90) is the loading control. Molecular weight ladder (kD) is shown on the right. **(B)** EAE progression in WT and GRIP1-cKO mice. Clinical scores (upper left) were evaluated at indicated DPI and plotted as the mean ± SEM of individual male (M) and female (F) mice (WT = 27; GRIP1-cKO = 22) from three independent experiments (Kruskal-Wallis test with Dunn’s multiple comparisons test). Mice were weighed at DPI0 and DPI20, and the fraction lost was compared (upper right; unpaired two-tailed Student’s *t* test). Mice at clinical scores ≥8 were killed; the Kaplan-Meier estimator was used to analyze survival (lower left; Mantel-Cox test). Disease incidence (%) and DPI of symptom onset in WT and GRIP1-cKO mice are shown (lower right). *, P < 0.05; **, P < 0.01. **(C)** At DPI20, blood was collected by cardiac puncture, and inflammatory cytokine levels were measured using CBA (WT = 10; GRIP1-cKO = 12 from two independent experiments; unpaired two-tailed Student’s *t* test). *, P < 0.05; **, P < 0.01.

As MG plays a central role in both MS and EAE, we first established a cell culture system to study and manipulate these cells ex vivo. We isolated primary MG from P0 neonatal mice and expanded mixed glial cultures of MG on the monolayer of astrocytes (see Materials and methods). MG were then purified on CD11b-coated beads and treated with proinflammatory LPS for 2 h in the absence or presence of either dexamethasone (Dex; a synthetic GC) or IFN-β, the two compounds clinically used to alleviate neuroinflammation in MS, followed by expression profiling using RNA sequencing (RNAseq). Of 963 LPS-regulated genes, 553 were induced; of those, 163 were downregulated by Dex and 115 were downregulated by IFN-β (Fig. S1 A, top). Interestingly, only a small group of 20 genes overlapped in the two datasets; that is, they were repressed by both Dex and IFN-β, but those encoding key proinflammatory cytokines *Tnf*, *Il1a*, *Il1b*, and *Il12b* were among them (Fig. S1 A, top right, underlined). We have previously established that GRIP1 mediates anti-inflammatory actions of GR in BM-derived primary MФ (BMMФ) by potentiating both activation of anti-inflammatory genes (e.g., *Tsc22d3*, *Dusp1*) and repression of proinflammatory ones such as *Tnf*, *Il1a*, and *Il1b* (Rollins et al., 2017). To examine the impact of GRIP1 loss in MG, we performed RNAseq analysis on the P0 MG derived in culture from the LysMCre^+/+^;GRIP1^fl/fl^ mouse strain (referred to as GRIP1-cKO) lacking GRIP1 in the myeloid lineage (Fig. 1 A and Fig. S1 B). From 1,403 LPS-responsive genes, 854 were induced, and approximately one-half of them (430) were repressed by Dex; yet, only 96 were downregulated by IFN-β (Fig. S1 A, bottom). As a result, the number of genes repressed by both Dex and IFN-β in GRIP1-cKO MG was down to 11 and no longer included *Tnf* and *Il12b* (Fig. S1 A, bottom right, underlined).

A quantitative assessment of the consequences of GRIP1 deletion on repression of proinflammatory genes by GR revealed that GRIP1 loss in MG modestly attenuated repression of *Nlrp3* (Fig. S1 B). In addition, a typical ISG, *Ifit1*, was less IFN-β responsive in GRIP1-cKO MG than WT (Fig. S1 B). Overall, the impact of GRIP1 deletion on representative genes of these two classes ex vivo resembled that seen in MФ. Of note, the effect of type I IFN on LPS-induced genes and the potential role of GRIP1 in this context have not been previously evaluated in any cell type.

### Myeloid cell–specific deletion of GRIP1 in vivo attenuates EAE

**Figure S2.**
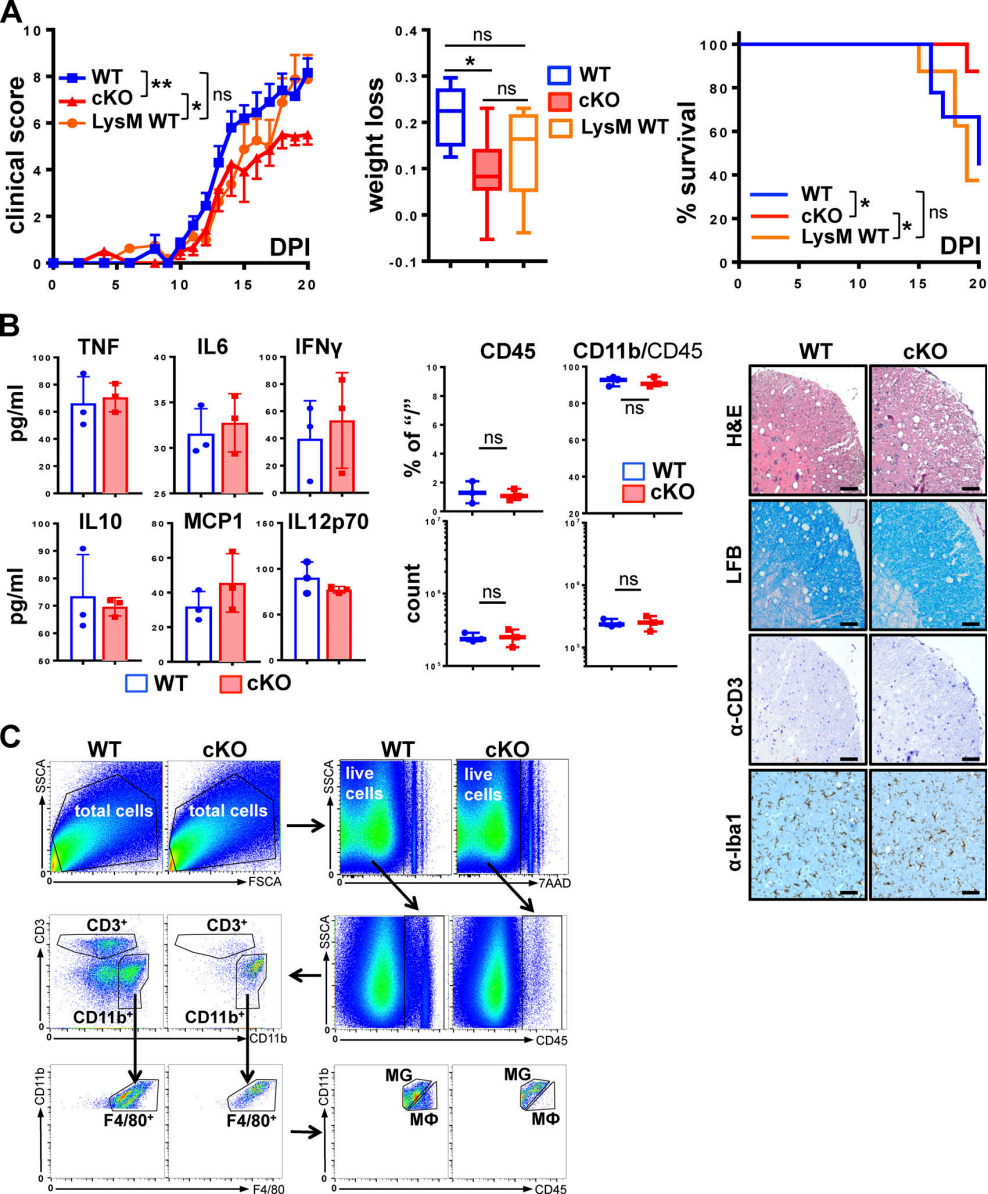
**Disease severity is independent of Cre expression, and homeostatic GRIP1-cKO mice display no apparent phenotype. (A)** Clinical scores were determined daily following EAE induction in eight WT, nine LysM WT, and seven cKO mice and plotted as mean ± SEM (Kruskal-Wallis test with Dunn’s multiple comparisons test at DPI20). The fraction of weight lost by DPI20 was assessed in WT, LysM WT, and cKO mice as in Fig. 1 B (Kruskal-Wallis test with Dunn’s multiple comparisons test at DPI20). Survival distribution was plotted via Kaplan-Meier curve and compared between strains as in Fig. 1 B (Mantel-Cox test). *, P < 0.05; **, P < 0.01. ns, nonsignificant. **(B)** Homeostatic age-matched WT and cKO mice (*n* = 3) were killed, and their blood was collected by cardiac puncture. Serum concentrations of indicated inflammatory cytokines were measured using CBA as in Fig. 1 C. Spinal cord lumbar sections from age-matched WT and cKO mice were analyzed as in Fig. 2 A by H&E staining for inflammatory foci, LFB staining for myelin, immunohistochemistry for CD3^+^ T cells, and Iba-1^+^ for MG and MФ. Scale bar is 100 µm. FACS analysis of leukocytes isolated from spinal cords of WT or cKO mice (*n* = 3) is plotted as a percentage of the gated parent population and total counts. **(C)** FACS gating strategy for all experiments. Cells were purified from spinal cords of WT and GRIP1-cKO mice with EAE and separated from myelin using Percoll gradient. From total selected cells, live (7-AAD^−^) cells were gated on. Of those, CD45^+^ cells were gated on as leukocytes and separated into two populations: CD3^+^ T cells and CD11b^+^ cells containing B cells, myeloid cells, and natural killer cells. The myeloid cell subpopulation expressing F4/80 was regated and divided into Cd11b^+^CD45^low^ MG and Cd11b^+^CD45^high^ MФ. FSCA, forward-scatter area; SSCA, side-scatter area.

Given extensive evidence for the transcriptional makeup of MG being determined by the local CNS environment (Gosselin et al., 2017), we reasoned that evaluating MG responses following 3-wk differentiation and expansion in culture may underestimate the impact of treatments, of GRIP1 deletion, or both on MG biology. Thus, to assess the role of GRIP1 in MG in vivo, we induced EAE in WT and GRIP1-cKO mice and monitored disease progression (see Materials and methods). In stark contrast to the endotoxin shock model, GRIP1-cKO mice displayed dramatically lower EAE scores than WT, which correlated with less weight loss and better survival with no significant difference in symptom onset time or incidence between groups (Fig. 1 B). Notably, there were no sex-specific differences in EAE severity between WT and GRIP1-cKO mice; that is, male and female GRIP1-cKO mice were similarly protected (Fig. 1 B). Importantly, the EAE-resistant phenotype of GRIP1-cKO was evident, regardless of whether the LysM-expressing (LysMCre^+/+^;GRIP^wt/wt^) or LysM-nonexpressing (LysMCre^−/−^;GRIP1^fl/fl^) strain was used as a WT control (Fig. S2 A). Further departing from the “cytokine storm” phenotype of the GRIP1-cKO in the endotoxin shock model (Chinenov et al., 2012; Rollins et al., 2017), the levels of signature T helper type 1 cell (Th1) cytokines TNF, IFN-γ, and IL-6 during EAE were reduced in the serum of GRIP1-cKO compared to WT mice, corresponding to their less severe systemic inflammatory response (Fig. 1 C), with no difference between genotypes seen at homeostasis (Fig. S2 B, left).

**Figure 2.**
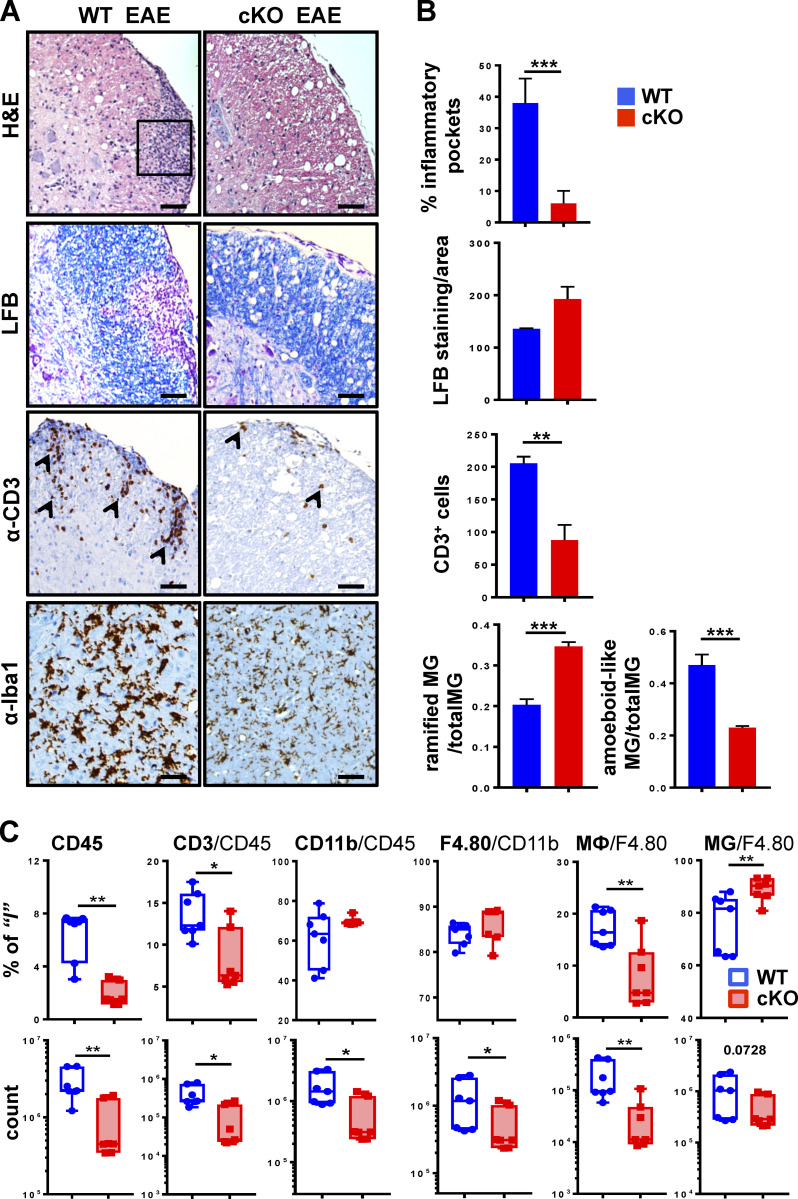
**Histological signs of EAE are attenuated in spinal cords of GRIP1-cKO mice. (A)** Spinal cord lumbar sections from WT and GRIP1-cKO mice at DPI20 from three independent experiments were analyzed by H&E staining for inflammatory foci (rectangle), LFB staining for myelin, and immunohistochemistry for CD3^+^ infiltrating T cells (arrowheads) and Iba-1^+^ MG (in parenchyma). Scale bars are 100 µm. **(B)** Quantification of slides from A for inflammation (percentage of inflammatory pockets), demyelination (LFB-stained myelin/area), CD3^+^ cells, and a number of ramified (with processes) and amoeboid-like (round-shaped with retracted processes) MG was performed as described in the Materials and methods section (unpaired two-tailed Student’s *t* test). **, P < 0.01; ***, P < 0.001. **(C)** FACS analysis of leukocytes isolated from spinal cords of WT or GRIP1-cKO mice at DPI20 is plotted as a percentage of gated parent population and total counts (Mann-Whitney *U* test). *, P < 0.05; **, P < 0.01.

Histological signs of MS and EAE are leukocyte infiltration, white matter damage, and demyelination of the CNS (Gibson-Corley et al., 2016; Pyka-Fosciak et al., 2018). We assessed these parameters in cervical, lumbar, and thoracic spinal cord segments from WT and GRIP1-cKO mice at homeostasis (Fig. S2 B, right) and during EAE (Fig. 2). H&E staining revealed a dramatically attenuated leukocyte infiltration in the CNS of GRIP1-cKO relative to WT, which correlated with a reduced number of infiltrating T lymphocytes (Fig. 2, A and B); Luxol fast blue (LFB) staining of myelin showed areas of local demyelination in the CNS of WT (Fig. 2, A and B). Functional states of MG can be defined morphologically; at homeostasis, MG are ramified and exhibit highly branched processes, whereas during inflammation, activated MG retract their processes and enlarge cell bodies due to organelle buildup and increased metabolic activity (Sominsky et al., 2018). Fig. 2, A and B, demonstrate Iba-1–positive MG located in the parenchyma of WT and GRIP1-cKO spinal cords with visibly enlarged, amoeboid-like MG in the WT compared with ramified, multiprocessed MG in the GRIP1-cKO. No histological differences between WT and GRIP1-cKO spinal cords were observed at homeostasis (Fig. S2 B, right).

Given that EAE is driven by both CNS-resident and CNS-infiltrating peripheral immune cells, we profiled the immune cell populations in the spinal cords of WT and GRIP1-cKO mice by flow cytometry at homeostasis (Fig. S2 B, middle) and following EAE induction (Fig. 2 C). Immune cells were sorted using common lineage-specific surface markers (see gating strategy in Fig. S2 C; Greter et al., 2015). We differentiated MG from MФ on the basis of expression level of CD45 (MG, CD45^low^; MФ, CD45^high^; Fig. S2 C) as described previously (Rangaraju et al., 2018; Sedgwick et al., 1991). Consistent with clinical scores and histopathology, although no difference in the CNS-resident immune cell number was seen between WT and GRIP1-cKO at homeostasis (Fig. S2 B, middle), during EAE, GRIP1-cKO accumulated fewer total leukocytes (CD45^+^), T lymphocytes (CD3^+^), and myeloid and B cells (CD11b^+^) in their CNS in counts and in percentage of initial population than did WT mice. Importantly, the strikingly lower number of infiltrating MФ in the CNS of GRIP1-cKO mice resulted in the apparently higher fraction of “less diluted” resident MG among the F4/80^+^CD11^+^ cells (Fig. 2 C). Together, these results demonstrate that, contrary to its inhibitory actions in other models of MФ-driven inflammation, myeloid cell GRIP1 plays a permissive role in the onset and/or progression of EAE.

### GRIP1 facilitates the neuroinflammatory “effector” stage of EAE

**Figure 3.**
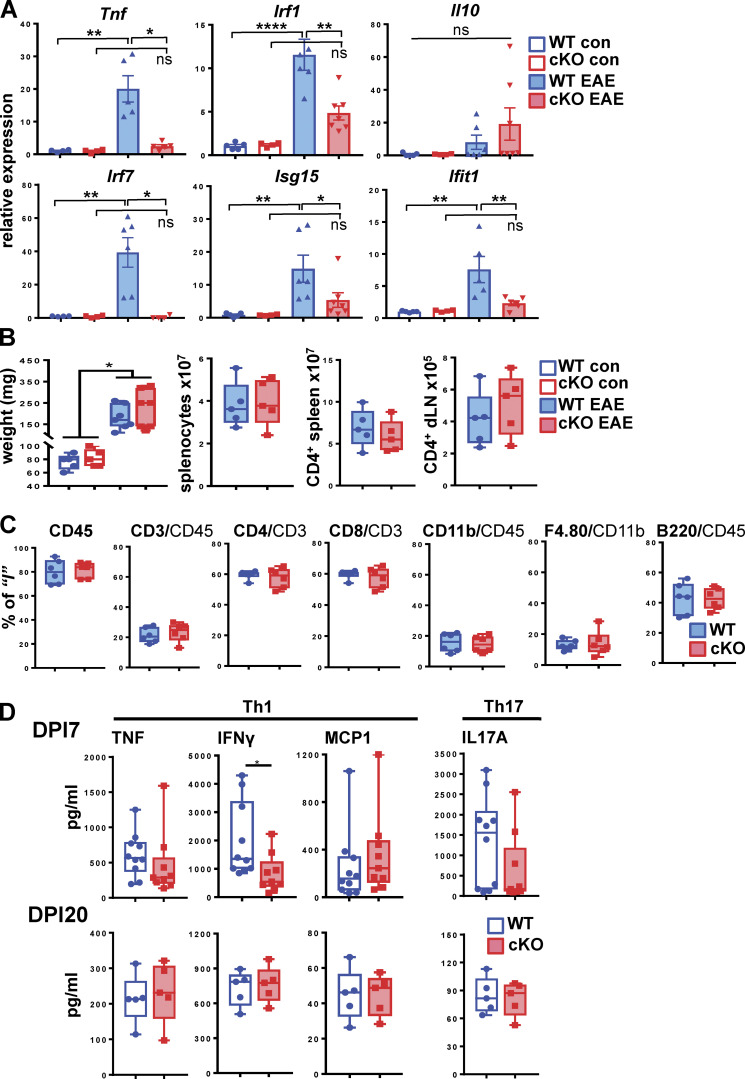
**GRIP1-cKO mice develop less brain inflammation than WT mice but a similar peripheral T cell response in vitro. (A)** Brains were harvested from control (WT = 5; GRIP1-cKO = 4) and EAE DPI20 (WT = 6; GRIP1-cKO = 6) mice from two independent experiments, and total RNA was extracted. Relative expression of the indicated genes was evaluated by RT-qPCR, normalized to that of the *Actb* housekeeping gene, and expressed relative to WT control (= 1; two-way ANOVA with Tukey’s multiple comparisons test). *, P < 0.05; **, P < 0.01; ****, P < 0.0005. ns, nonsignificant. **(B)** Spleens were collected from WT and GRIP1-cKO control mice (*n* = 5 each) and EAE DPI20 mice (*n* = 7 each) from one experiment, and their weights (in mg) were compared (Kruskal-Wallis test with Dunn’s multiple comparisons test; *, P < 0.05). Numbers of splenocytes and CD4^+^ cells isolated from spleens and dLNs were quantified by FACS analysis. **(C)** FACS analysis of leukocytes isolated from spleens of WT and GRIP1-cKO mice at DPI20 is plotted as a percentage of the gated parent population. **(D)** Spleens were collected from WT and GRIP1-cKO mice at DPI7 (WT = 10; GRIP1-cKO = 9 from two independent experiments) and DPI20 (*n* = 5 each from one experiment). CD4^+^ T cells were isolated and restimulated with MOG_35–55_ in vitro, and the indicated Th1 and Th17 secreted cytokines were quantified using CBA (unpaired two-tailed Student’s *t* test). *, P < 0.05.

**Figure S3.**
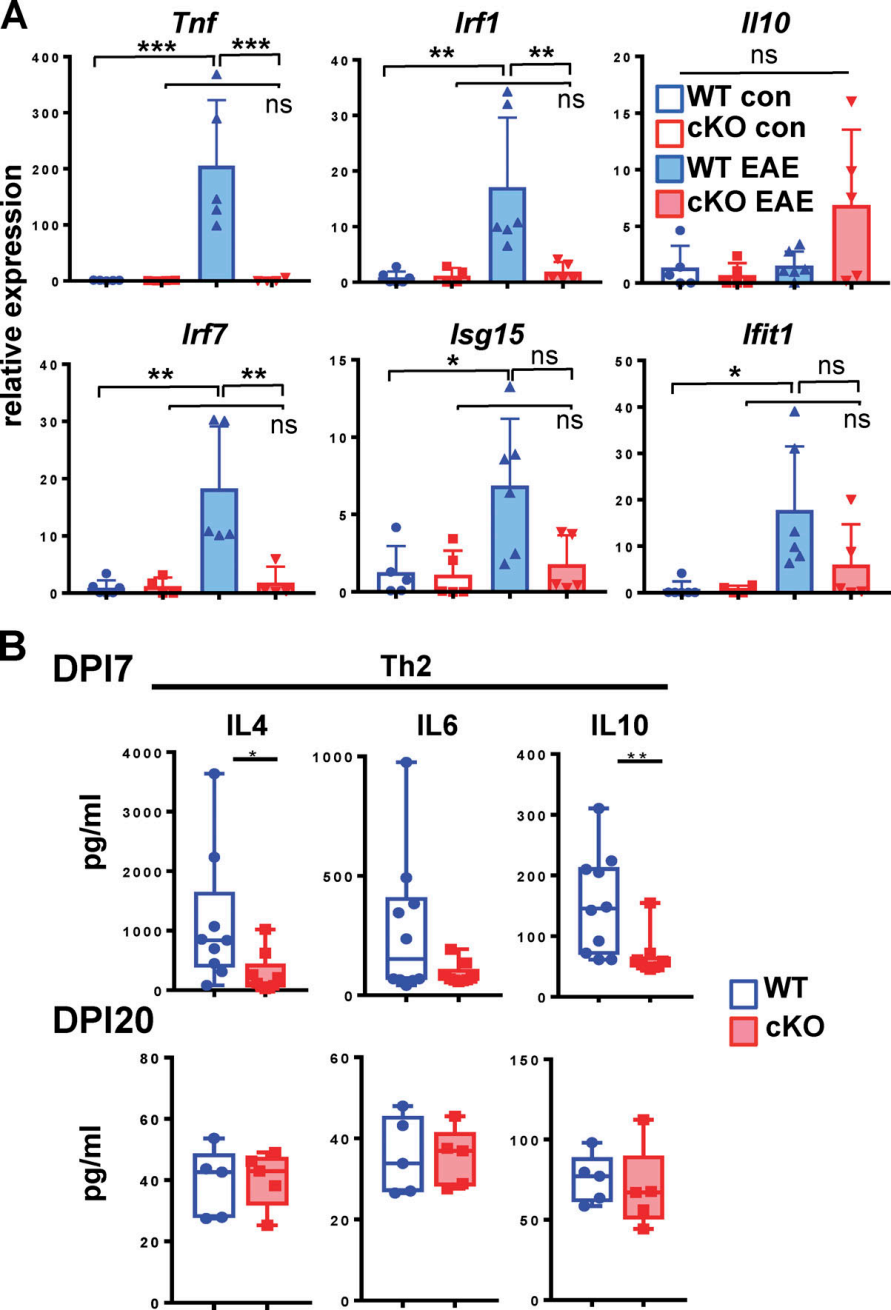
**GRIP1-cKO mice develop less spinal cord inflammation and attenuated early peripheral Th2 T cell response in vitro*.* (A)** Spinal cords were harvested from control (WT = 6; GRIP1-cKO = 5) and EAE DPI20 (WT = 6; GRIP1 cKO = 6) mice, and total RNA was extracted. Relative expression of the indicated genes was evaluated by RT-qPCR, normalized to that of the *Actb* housekeeping gene, and expressed relative to WT control (=1; two-way ANOVA with Tukey’s multiple comparisons test). *, P < 0.05; **, P < 0.01; ***, P < 0.005. ns, nonsignificant. **(B)** Spleens were collected at DPI7 (WT = 10; GRIP1-cKO = 9; two independent experiments) and DPI20 (*n* = 5 each from one experiment). CD4^+^ T cells were isolated, then restimulated with MOG_35–55_ in vitro, and the indicated Th2-secreted cytokines were quantified using CBA (unpaired two-tailed Student’s *t* test). *, P < 0.05; **, P < 0.01.

To determine the hallmarks of EAE pathogenesis that were sensitive to GRIP1 deletion, we evaluated the expression of inflammatory mediators in the CNS of control and EAE WT and GRIP1-cKO mice. Although no genotypic differences were observed in the level of any transcripts measured at homeostasis, there was a significant accumulation of proinflammatory *Tnf* but not anti-inflammatory *Il10* during EAE in the brains and spinal cords of WT mice, and this effect was greatly attenuated in GRIP1-cKO (Fig. 3 A and Fig. S3 A, top). Unexpectedly, several established components of the type I IFN network (*Irf1*, *Irf7*,* Isg15*, and *Ifit1)* were dramatically upregulated during EAE specifically in the WT CNS (Fig. 3 A and Fig. S3 A). Even though this result was consistent with the requirement for GRIP1 in the IFN pathway, a pronounced IFN signature in mice with more severe pathology argues against type I IFN serving a solely protective role in EAE/MS.

In principle, GRIP1 either can act on the periphery to facilitate an immune response and generation of myelin-reactive autoimmune T cells during the inductive phase or, alternatively, can contribute to myeloid cell–driven neuroinflammation during the effector phase. To assess the potential contribution of GRIP1 to each stage of the disease, first, we harvested peripheral immune organs—the spleens and draining LNs (dLNs)—from WT and GRIP1-cKO mice with or without EAE and evaluated their gross morphology. Although the size of the spleens increased significantly during EAE compared to that at homeostasis, there was no genotypic difference in the size of the spleens, number of splenocytes, or number of CD4^+^ T cells (Fig. 3 B). In addition, flow cytometry performed on the spleens of WT and GRIP1-cKO mice with EAE revealed similar percentages of leukocytes (CD45^+^), lymphocytes (CD3^+^), CD4^+^ or CD8^+^ T cells, B220^+^ B cells, and CD11b^+^ myeloid cells in the two genotypes (Fig. 3 C).

We next isolated CD4^+^ T cells from spleens and dLNs of WT and GRIP1-cKO mice at day 7 postimmunization (DPI7) or DPI20, restimulated them with myelin oligodendrocyte glycoprotein (MOG) peptide in vitro for 72 h, and analyzed the production of Th1 and Th17 cytokines implicated in MS pathology as well as the EAE model. Of those, we observed slightly reduced levels of IFN-γ produced by CD4^+^ T cells from GRIP1-cKO mice at DPI7 only, whereas the levels of TNF, MCP1, and IL-17A were identical in the two genotypes at both time points (Fig. 3 D). Interestingly, at DPI7, restimulated CD4^+^ T cells isolated from WT mice produced more IL-4 and IL-10 than the ones from GRIP1-cKO mice, but these differences were abrogated by DPI20 (Fig. S3 B). This result does not definitively establish the T cell subtype mediating EAE in our model, but it illustrates a transiently attenuated Th2 CD4^+^ T cell signature in the GRIP1-cKO mice compared to WT mice.

**Figure 4.**
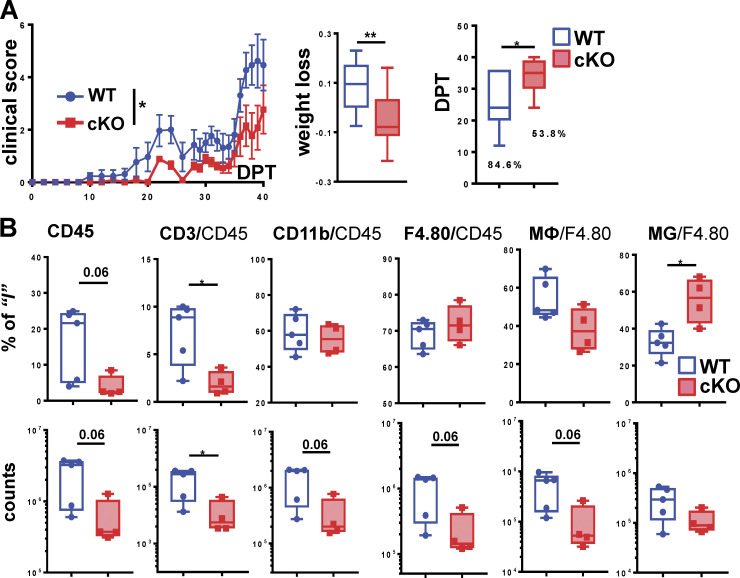
**GRIP1 contributes to the neuroinflammatory phase of EAE. (A)** Passive EAE was induced in WT and GRIP1-cKO mice (*n* = 13 each from two independent experiments), and clinical scores (left) were measured and plotted daily as mean ± SEM (Mann-Whitney *U* test). The fraction of weight lost between days post-transfer (DPT) 0 and 40 (middle) was compared using an unpaired two-tailed Student’s *t* test. The incidence of disease (%) is shown; time of symptom onset (right) was compared using the Mann-Whitney test. *, P < 0.05; **, P < 0.01. **(B)** FACS analysis of leukocytes isolated from spinal cords of five WT and four GRIP1-cKO mice (two independent experiments) at DPT40 is plotted as a percentage of the gated parent population and total counts (unpaired two-tailed Student’s *t* test). *, P < 0.05.

To definitively determine the stage of EAE during which GRIP1 contributes to disease, we used a passive EAE model in which the inductive and effector phases are uncoupled from each other. Following EAE induction in WT female donor mice, CD4^+^ T cells were collected from their spleens and dLNs at DPI10, Th1 polarized in vitro (see Materials and methods), and injected into recipient WT and GRIP1-cKO mice along with pertussis toxin (PTX). Despite expected lower clinical scores in this passive model, WT mice still developed more severe disease, which, strikingly, occurred earlier and in a greater number of animals than it did in the GRIP1-cKO mice (Fig. 4 A). Consistently, GRIP1-cKO mice lost less weight and displayed less immune cell infiltration of the CNS (Fig. 4, A and B). Together, these results demonstrate that myeloid cell GRIP1 facilitates the effector neuroinflammatory phase of EAE.

### Transcriptomic consequences of myeloid cell–specific GRIP1 deletion in the CNS

**Figure 5.**
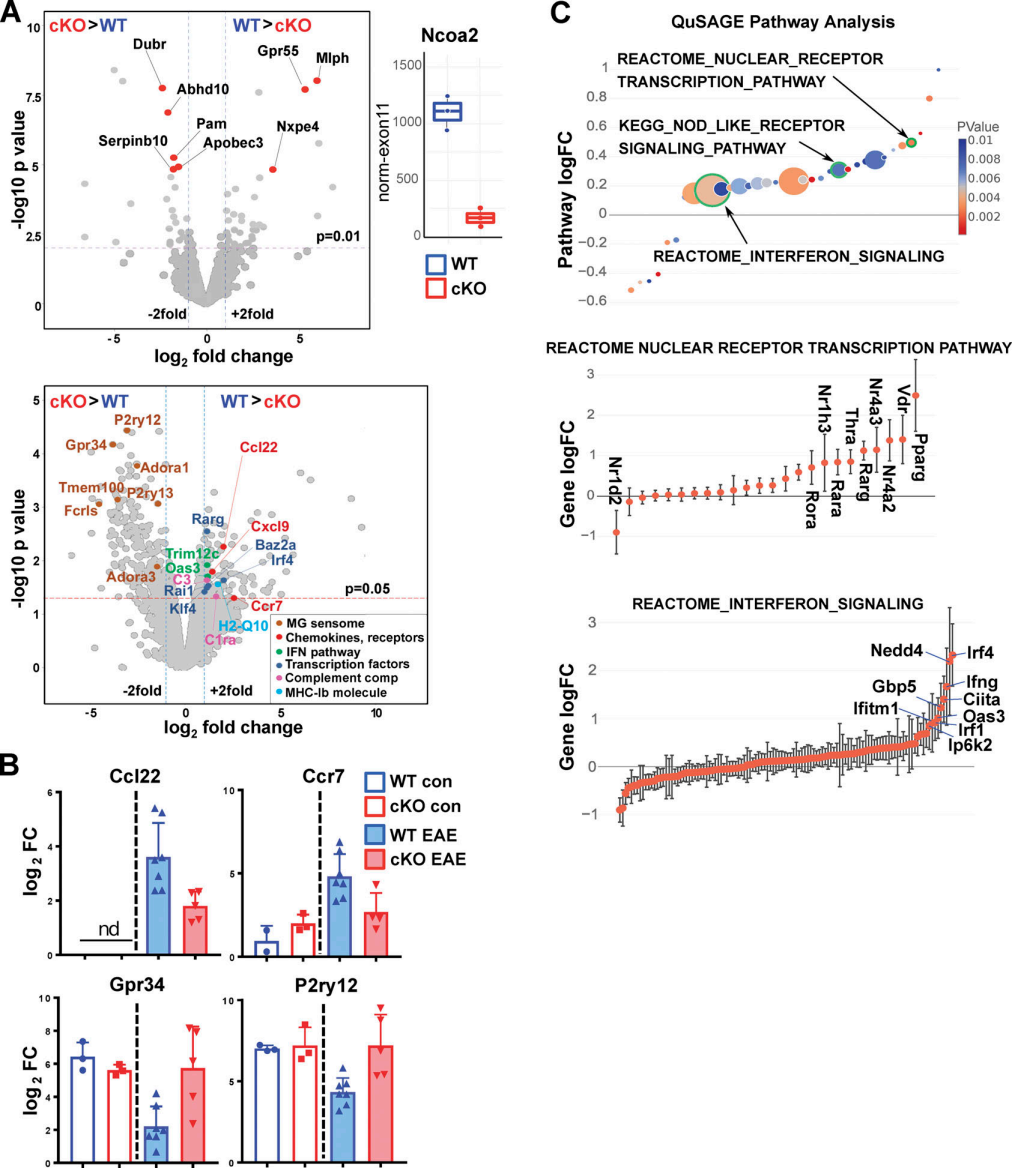
**Transcriptomic profiling of myeloid cells from WT and GRIP1-cKO mice during homeostasis and EAE. (A)** Myeloid gene expression was modeled as batch-corrected averages of gene expression in MФ (CD45^high^; *n* = 2–4) and MG (CD45^low^; *n* = 3). Top left: Volcano plot shows differentially expressed genes (red) in WT versus GRIP1-cKO (*n* = 3) MG from homeostatic mice (log_2_ fold change (log_2_FC) = 1; unadjusted P value = 0.01). Top right: Library size-normalized read counts in WT and GRIP1-cKO mice that mapped to Ncoa2 (GRIP1) exon 11 deleted by the Ncoa2 targeting construct (Gehin et al., 2002; *n* = 3). Bottom: Volcano plot shows differentially expressed genes in CD45^+^Cd11b^+^ activated myeloid cells isolated from spinal cords of WT and GRIP1-cKO mice with EAE (*n* = 7 and 5, respectively; log_2_FC = 1; unadjusted P = 0.05). Selected genes upregulated in EAE WT (WT > cKO) are shown on the right and highlighted in colors corresponding to their functional groups (boxed in lower right). Genes with higher expression in EAE GRIP1-cKO (cKO > WT) are shown on the left, and seven genes highlighted in brown represent the homeostatic MG “sensome.” **(B)** The RNAseq expression levels of select genes from A in WT and GRIP1-cKO homeostatic and EAE mice. **(C)** QuSAGE of differentially expressed genes from WT and GRIP1-cKO mice with EAE. Differentially expressed pathways (unadjusted P < 0.01) from MsigDB C2 set (Broad Institute) are shown. The size of the circles is proportional to the number of genes in the pathway, and the color is proportional to the P value. Genes for the nuclear receptor transcription pathway (middle) and IFN signaling (bottom) from the REACTOME database are plotted as logFC ± SD and ordered by the logFC. KEGG, Kyoto Encyclopedia of Genes and Genomes.

GRIP1 is a broadly acting transcriptional coregulator whose role in MS/EAE or in MG at any state has never been investigated. To begin to identify the GRIP1-dependent transcriptome changes leading to neuroinflammation, we performed bulk RNAseq analysis on CD45^+^Cd11b^+^ myeloid cells isolated from spinal cords of WT and GRIP1-cKO mice. Consistent with a lack of overt phenotype in our conditional GRIP1-deficient mice (Coppo et al., 2016; Rollins et al., 2017), at homeostasis, a CD45^+^Cd11b^+^ CNS myeloid cell population composed principally of MG displayed no significant transcriptomic differences between WT and GRIP1-cKO mice (Fig. 5 A, upper panel; GRIP1 deletion efficiency is shown on the right as normalized read counts across “floxed” exon 11 of the *Ncoa2* gene). In contrast, more heterogeneous activated CD45^+^Cd11b^+^ cells during EAE (Fig. S2 C and Fig. 2 C) presented distinct transcriptomic signatures in WT versus GRIP1-cKO mice. Indeed, genes upregulated in WT (Fig. 5 A, lower panel, and Fig. 5 B), such as chemokines and chemokine receptors (*Ccl22*, *Ccr7*), antigen presentation molecule (*H2-q10*), components of complement (*C3*, *C1ra*), and type I IFN (*Trim12c*, *Oas3*) pathways, are indicative of inflammation and EAE pathogenesis (Belikan et al., 2018; Salter and Stevens, 2017; Scheu et al., 2017). Interestingly, a pool of genes downregulated in WT mice during EAE but persisting in GRIP1-cKO mice (e.g., *Gpr34*,* P2ry12*; Fig. 5 A, lower panel, and Fig. 5 B) are homeostatic genes referred to as the MG “sensome” (Hickman et al., 2013), which controls chemotaxis and tissue repair (Lou et al., 2016).

To identify physiologically relevant pathways differentially active in myeloid cells from WT and GRIP1-cKO spinal cords during EAE, we performed quantitative set analysis of gene expression (QuSAGE; Yaari et al., 2013), a gene set enrichment analysis–like Bayesian method that provides better accounts for intergene correlations than classic gene set enrichment analysis. QuSAGE determines pathway-wide expression (pathway activity) by combining probability density functions for individual gene expression using numerical convolution. Several pathways were expressed at higher levels in the WT CNS myeloid cells, including NODE-like receptor signaling pathways (Kyoto Encyclopedia of Genes and Genomes) and a nuclear receptor transcription pathway (REACTOME), including Nr4a2 (Nurr1) and Nr4a3 (Nor-1; Fig. 5 C). Remarkably, several key genes of the IFN axis (IFN signaling pathway [REACTOME]), including *Irf4*, *Irf1*,* Ifng*,* Ifitm1*,* Gbp5*, and *Oas3*, were also expressed at higher levels in the WT (Fig. 5 C), in accord with whole-brain and spinal cord quantitative PCR (qPCR) data (Fig. 3 A and Fig. S5 A) and with a demonstrated coactivator role for GRIP1 in type I IFN network in MФ (Flammer et al., 2010; Reily et al., 2006). Collectively, these data demonstrate a failure to upregulate inflammatory and type I IFN pathways and persistence of homeostatic signature in GRIP1-cKO myeloid cells; however, it could potentially stem from the role of GRIP1 in MG, MФ, or both.

**Figure 6.**
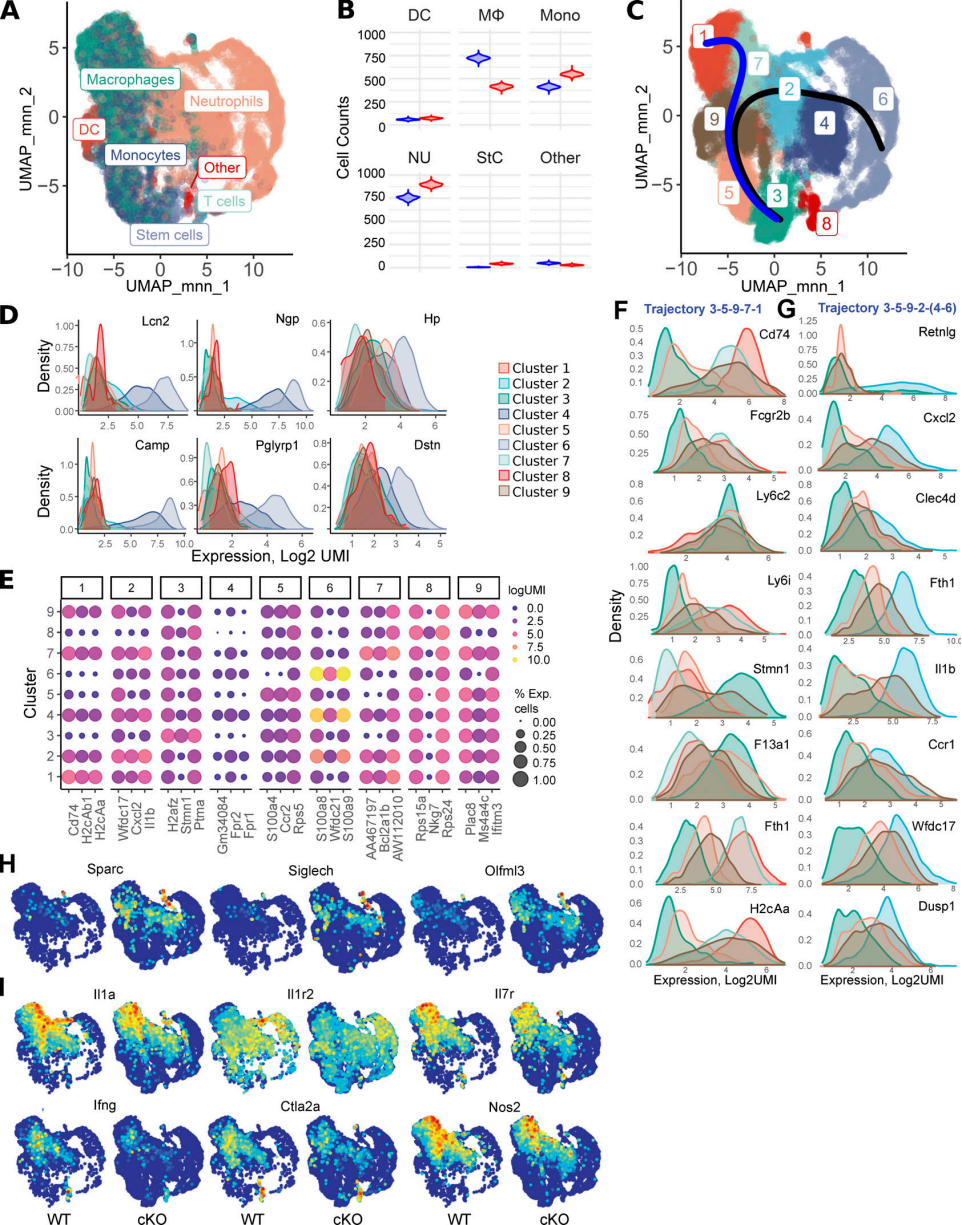
**scRNAseq of spinal cord myeloid cells isolated from WT and GRIP1-cKO mice at EAE DPI20. (A)** singleR automated cell type assignment for 20,376 spinal cord–derived myeloid cells. **(B)** Bootstrapping analysis (see Materials and methods) of singleR cell type (from A) distribution, stratified by genotype: WT = blue; GRIP1-cKO = red. **(C)** Louvain clustering results and singleR trajectory inference for 20,376 spinal cord–derived myeloid cells mapped onto a UMAP plot. **(D)** Expression of neutrophilic markers, stratified by Louvain clusters. The color of expression profiles corresponds to Louvain clusters in C. **(E)** Bubble plot of top three cluster-specific markers (from C), with the circle size representing the percentage of expressing cells and the color corresponding to logUMI. **(F and G)** Expression of select genes along the (F) 3–5-9-7-1 (C, blue line) and (G) 3–5-9-2-4-6 (C, black line) trajectories. The color of expression profiles corresponds to Louvain clusters in C. **(H and I)** Expression of homeostatic MG (H) or inflammatory (I) markers mapped onto a UMAP plot, stratified by genotypes. DC, dendritic cells.

**Figure S4.**
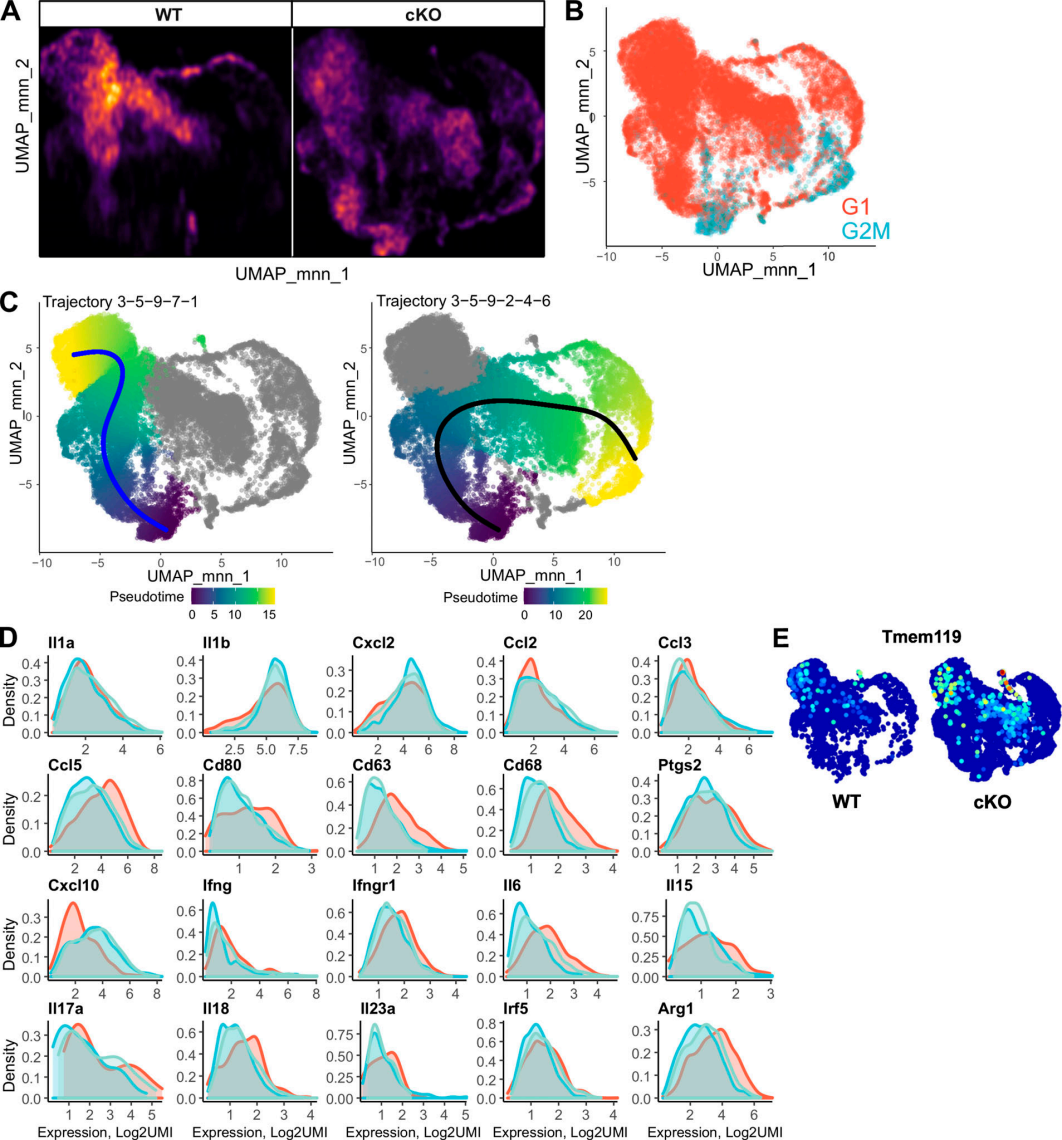
**scRNAseq of spinal cord myeloid cells from WT and GRIP1-cKO mice at EAE DPI20. (A)** Bootstrapping analysis of cell densities in the UMAP1–UMAP2 coordinates, stratified by genotypes. 2,000 cells were randomly sampled for each genotype, and the two-dimensional density matrix was calculated for an 800 × 800 binned matrix. The sampling was performed 500 times, and the average density for each bin was computed and plotted. **(B)** Automated cell cycle stage assignment for 20,376 spinal cord–derived myeloid cells. **(C)** singleR trajectory inference for 20,376 spinal cord–derived myeloid cells mapped onto a UMAP plot and colored by pseudo-time. Blue and black lines represent fitted PC curves. **(D)** Expression of MΦ markers in MΦ-like clusters 1 (red), 2 (blue), and 7 (light green). The color of expression profiles corresponds to Louvain clusters in Fig. 6 C. **(E)** Expression of homeostatic MG markers mapped onto a UMAP plot, stratified by genotypes.

To dissect the contribution of resident versus infiltrating myeloid cells to EAE pathogenesis, we performed single-cell RNAseq (scRNAseq) analysis of all myeloid CD45^+^CD11b^+^ cells from WT and GRIP1-cKO spinal cords at the peak of EAE (DPI20). After filtering out low-quality barcodes (see Materials and methods), we analyzed 20,376 cells (6,427 WT and 11,949 cKO) expressing 11,093 genes. Automated cell type assignment with singleR yielded four major clusters—“monocytes,” “MФ,” “dendritic cells,” and “neutrophils” (Fig. 6 A and Table S1)—and a large number of minor clusters composed predominately of lymphoid cell impurities that were collected during the cell sorting and had the same location in uniform manifold approximation and projection (UMAP) coordinates (Fig. 6 A). Because of an unbalanced group size, we performed a bootstrapping analysis to determine the associations between genotype and singleR cell types. We counted cell types of 2,000 cells that were sampled with the replacement from each genotype with 500 repeats (Fig. 6 B and Fig. S4 A). This analysis indicated that singleR monocytes and neutrophils were more common in the cKO, whereas MФ were overrepresented in the WT. There was a substantial overlap between singleR cell types, suggesting either the presence of cell subpopulations or different differentiation/activation states. To separate these states, we performed Louvain graph–based community clustering that yielded nine clusters (Fig. 6 C and Table S2). Cluster 8 corresponded to singleR lymphoid cell–enriched group (Fig. 6 A; “Others,” “T cells”), whereas cluster 6 was highly enriched with canonical neutrophilic markers (Fig. 6, A, D, and E). Cluster 3 is enriched in proliferation markers (Fig. 6 E, Fig. S4 B, and Table S4). Slingshot trajectory analyses anchored on cluster 3 (see Materials and methods) identified two main trajectories (3-5-9-7-1 and 3-5-9-2-4-6) bifurcating at cluster 9 (Fig. 6 C and Fig. S4 C). The analysis of genes differentially expressed along trajectories suggested that the 3-5-9-7-1 trajectory likely corresponds to monocyte-to-MФ transitions. Conversely, clusters 2-4-6 exhibit an increasing gradient of expression of neutrophilic markers (Fig. 6 E; *S100a8*,* S100a9*), suggesting that clusters 4 and 2 contain a decreasing admixture of neutrophils from cluster 6. Cluster 3 expresses monocytic markers at high levels (Fig. 6 F; *Ly6c2*,* F13a1*,* Stmn1*) and activated MФ/MG markers at low levels (Fig. 6, F and G; *Cd74*,* Fth1*,* Fcgr2b*,* H2-Aa*,* Il1b*) that reciprocally change along the trajectories. MФ-like clusters (1, 7, and 2) contain either different proportions of MФ/MG, different activation states, or an admixture of other cell types (e.g., oligodendrocyte precursors; Table S3). Although expression distributions for activated MФ/MG markers are broadly comparable in these clusters (Fig. S4 D), differential expression analysis between WT and cKO stratified by Louvain clusters revealed that clusters 1, 7, 2, and 4 expressed markers of homeostatic MG at higher levels in the cells from cKO mice (Fig. 6 H, Fig. S4 E, and Table S5; *Sparc*,* Siglech*,* Olfml3*, and *Tmem119*). Cluster 2 contained the largest percentage of cells expressing homeostatic MG markers. Conversely, many markers of activated inflammatory MФ were upregulated in these clusters in the WT cells including *Il1a*,* Il1r2*,* Il7r*,* Ifng*,* Ctla2s*, and* Nos2* (Fig. 6 I and Table S5).

### GRIP1 mediates the therapeutic effect of IFN-β

GCs and IFN-β are a standard of care for patients with RRMS. Consistently, genetic deletion of IFN-β or GR in mice increases EAE severity, whereas exogenous administration of GCs or IFN-β prevents relapses and ameliorates disease symptoms (Goodin, 2014; Vosoughi and Freedman, 2010; Wingerchuk and Carter, 2014). Given that GRIP1 is a cofactor for both GR and IRFs, the effectors of GC and type I IFN signaling, respectively, we examined whether myeloid cell–specific GRIP1 deletion impacts therapeutic potency of these agents in our mice.

**Figure S5.**
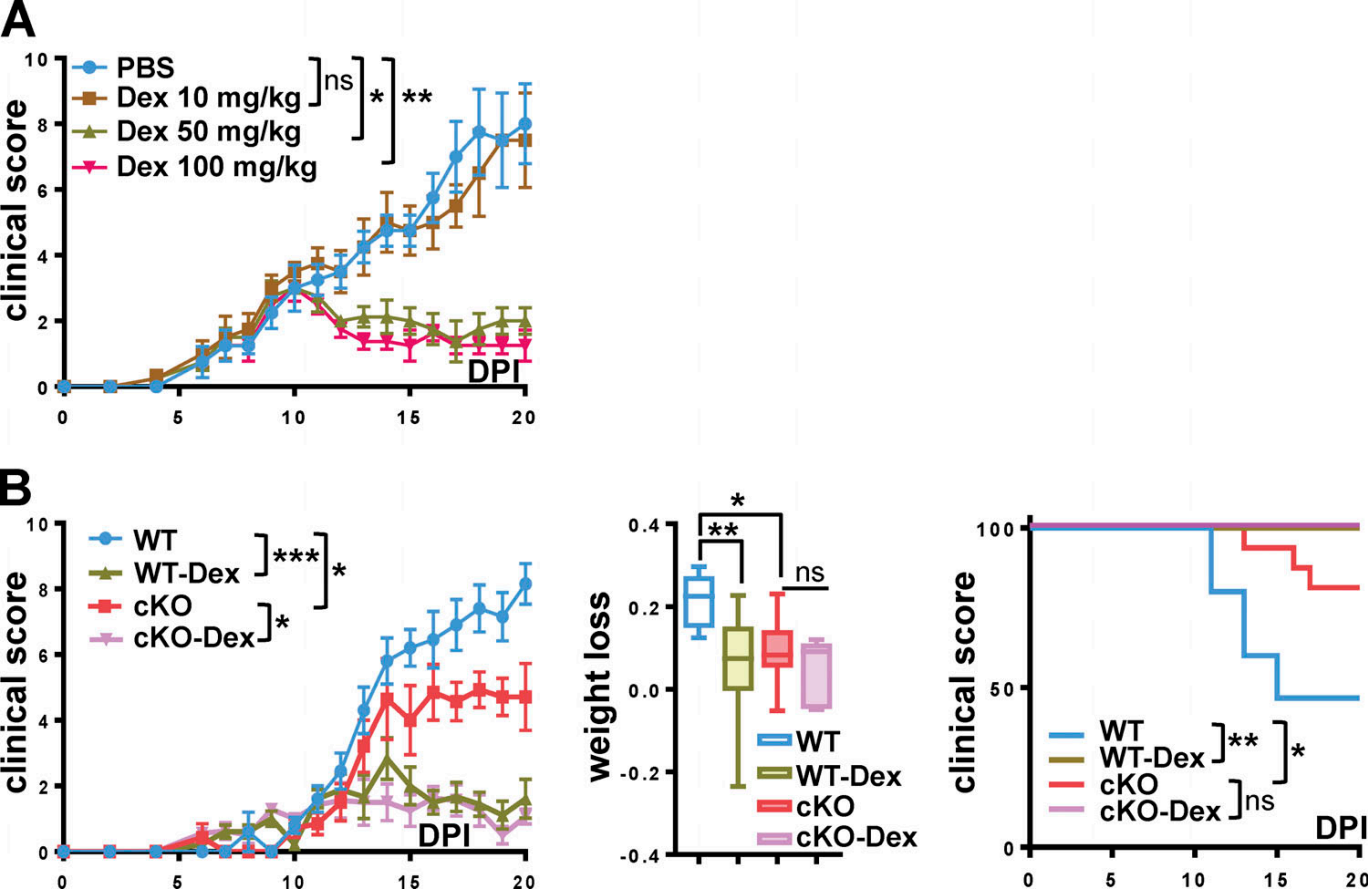
**Dex treatment reverses EAE in WT and cKO mice. (A)** WT mice were injected with PBS or the indicated amounts of Dex i.p. daily at EAE symptom onset (DPI10), and clinical scores were determined daily as mean ± SEM (*n* = 4 for each group; Kruskal-Wallis test with Dunn’s multiple comparisons test at DPI20). **(B)** At EAE DPI10, WT and GRIP1-cKO mice were divided into two groups that received either PBS or 50 mg/kg Dex i.p. daily. Clinical scores were measured daily as mean ± SEM (WT = 10, cKO = 4; Kruskal-Wallis test with Dunn’s multiple comparisons test at DPI20). Fraction of weight lost by DPI20 was measured in WT and cKO mice treated as above (unpaired two-tailed Student’s *t* test). The survival distribution in each group plotted via Kaplan-Meier curve was evaluated using the Mantel-Cox test as in Fig. 1 B (WT = 15, cKO = 10, WT Dex = 10, and cKO Dex = 8 mice from two independent experiments). *, P < 0.05; **, P < 0.01; ***, P < 0.001. ns, nonsignificant.

**Figure 7.**
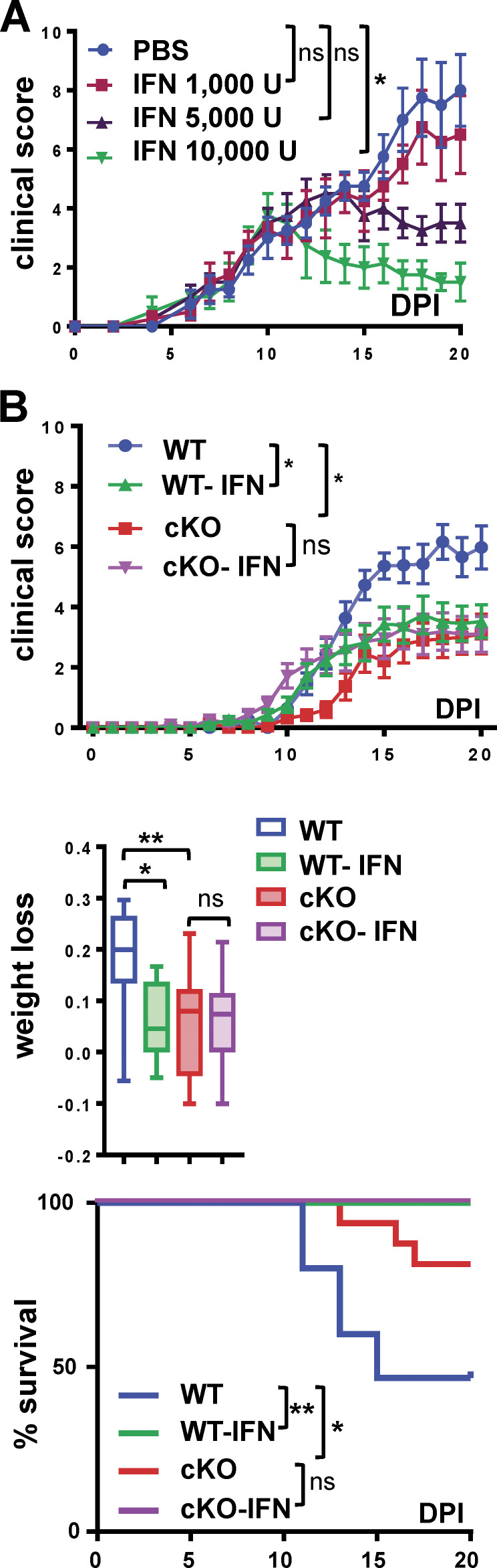
**GRIP1 mediates IFN-β therapeutic effect in EAE. (A)** Starting on EAE DPI10, WT mice were injected with either PBS or the indicated doses of IFN-β i.p. daily, and clinical scores were measured daily (mean ± SEM; *n* = 4 for each group; Kruskal-Wallis test with Dunn’s multiple comparisons test). **, P < 0.01. **(B)** Starting on DPI10, mice were injected as in A with PBS (WT = 18, KO = 13; two independent experiments) or 10,000 U of IFN-β (WT-IFN = 15, KO-IFN = 18; two independent experiments), and clinical scores were plotted daily as mean ± SEM (Kruskal-Wallis test with Dunn’s multiple comparisons test at DPI20). The fraction of weight lost was compared between the four groups of mice as described in Fig. 1 B (Kruskal-Wallis test with Dunn’s multiple comparisons test). The survival distribution for each group was plotted via Kaplan-Meier curve (Mantel-Cox test). *, P < 0.05; **, P < 0.01. ns, nonsignificant.

Both Dex (50–100 mg/kg; Fig. S5 A) and recombinant IFN-β (5,000–10,000 U; Fig. 7 A) reduced clinical scores of EAE in WT mice in a dose-dependent manner. Next, WT and GRIP1-cKO mice were administered PBS vehicle, 50 mg/kg Dex, or 10,000 U of IFN-β per mouse, doses that proved effective in WT mice, at symptom onset (clinical score, 2.0), i.p. daily for 10 d. Compared with PBS-treated controls, Dex therapy fully reversed EAE progression, including clinical scores, weight loss, and mortality, in both genotypes, indicating that GRIP1 did not mediate the therapeutic effect of Dex in this model (Fig. S5 B). Intriguingly, IFN-β treatment that dramatically reduced clinical scores, weight loss, and lethality of WT mice failed to elicit any improvement in the GRIP1-cKO mice (Fig. 7 B), demonstrating that myeloid cell GRIP1 was required for IFN-β efficacy in the neuroinflammation model.

## Discussion

### The role of GRIP1 in neuroinflammation

MS is a complex disease involving an interplay between innate and adaptive immune cells in the periphery and in the CNS. Notably, myeloid cells (MG and MФ) play a dual role in the pathogenesis: on the one hand, they promote neuroinflammation through antigen presentation, cytokine and chemokine secretion, and active demyelination; on the other, they help resolve it by clearing debris, secreting neurotrophic factors, and facilitating tissue repair (Herz et al., 2017). This high functional plasticity relies on precise environment-dependent transcriptomic reprogramming. Thus, identifying potential regulators that mediate each process is mandatory for understanding how each cell type contributes to or counteracts disease. Here, we propose that GRIP1 is an as yet unappreciated transcriptional coregulator driving myeloid cell–dependent neuroinflammation.

We show that mice lacking GRIP1 in myeloid cells had lower clinical scores that correlated with diminished immune cell infiltration and a reduced proinflammatory signature in myeloid cells of the CNS in vivo. This phenotype was unexpected, given well-documented anti-inflammatory actions of GRIP1 in MФ. However, those studies focused on peripheral MФ in the peritoneum or metabolic tissues, whereas CNS MФ/MG function in a unique environment and are exposed to myelin debris, other glial cells including astrocytes, and neurons, each potentially impacting GRIP1 behavior. A lack of CNS-specific environmental inputs may also account for the peripheral MФ-like responses to GRIP1 deletion in our in vitro differentiated P0 MG in culture. A tissue-specific transcriptional signature of specialized human and mouse MФ, such as MG, is reportedly “erased” ex vivo fairly rapidly as they dedifferentiate into a more “generic” myeloid cell (Gosselin et al., 2017). Moving forward, it will be critical to develop alternative methods for MG culture that do not involve extended expansion protocols.

Importantly, the role of GRIP1 in MG has not been studied and could in principle be opposite that in MФ. Indeed, the reciprocal functions in MФ versus MG in EAE pathogenesis have been shown previously for TNFR2. Specifically, TNFR2 ablation in MG induced an early-onset disease with increased leukocyte infiltration and T cell activation and demyelination, whereas its depletion in monocytes/MФ suppressed EAE, impaired T cell activation, and reduced demyelination (Gao et al., 2017). Perhaps GRIP1 could potentiate inflammatory gene transcription in MG, with its depletion conferring a neuroprotective homeostatic MG state.

Transcription factors that mediate GRIP1 proinflammatory properties in WT mice remain to be identified; however, upregulation of genes encoding several nuclear receptors for which GRIP1 is a known coactivator is notable in this regard. QuSAGE pathway analysis revealed the upregulation of *Nr4a2* (Nurr1) and *Nr4a3* (NOR-1) transcripts in myeloid cells from spinal cords of WT EAE mice relative to GRIP1-cKO mice. Members of the *Nr4a* subfamily of orphan nuclear receptors are emerging as key regulators of inflammation, with both anti- and proinflammatory activities reported (Koenis et al., 2018; Pei et al., 2006; Raveney et al., 2013; Rothe et al., 2017). They are induced by NF-κB within 30 min of exposure to pleiotropic inflammatory stimuli, including LPS, cytokines, and peptide hormones in immune cells (myeloid, T cells) and other tissues involved in chronic inflammation (synovial tissue, atherosclerotic lesions). *Nr4a* receptors in turn control their target genes by binding either to a specific octamer DNA sequence known as the NGFI-B response element as monomers or to palindromic sequences termed the Nur response element as homo- or heterodimers and activating or repressing transcription (Kurakula et al., 2014; Murphy and Crean, 2015; Rodríguez-Calvo et al., 2017). In support of an anti-inflammatory role of these receptors, Nur77 (encoded by *Nr4a1*) and Nurr1 have been linked to a homeostatic phenotype in MФ (Bonta et al., 2006; Hanna et al., 2012; Ipseiz et al., 2014; Koenis et al., 2018; Mahajan et al., 2015). Moreover, in glial cells, Nurr1 inhibits expression of NF-κB–induced inflammatory neurotoxic mediators by recruiting the CoREST corepressor complex to the NF-κB binding sites (Saijo et al., 2009). On the other hand, Nurr1 was shown to be upregulated in peripheral blood T cells of MS patients and to drive transcription of proinflammatory *Il17*, *Ifng*, and *Il2* in mice with EAE (Doi et al., 2008); it also reportedly participates in Th17 cell maturation and controls their ability to produce IL-21 (Raveney et al., 2013). In addition, Nur77 has been shown to promote the expression of inflammatory genes such as *MARCKS*, *IKBKE*, and *MAP3K14* in LPS-activated MФ (Pei et al., 2006), whereas Nurr1 activates matrix metalloproteinase genes in synoviocytes (Mix et al., 2007). Thus, GRIP1 could facilitate inflammatory gene expression in myeloid cells during EAE in conjunction with Nurr1 and NOR-1. Importantly, retinoid X receptor, the Nr4a heterodimeric partner, was also upregulated in the CNS myeloid cells of our WT EAE mice.

Neuroinflammation involves functional interactions between myeloid cells and surrounding cells of the CNS. Indeed, astrocytes are key players in CNS inflammation (Brambilla et al., 2014; Rothhammer et al., 2018), and activated MG, in EAE and MS, drive astrocyte activation and neurotoxicity (Liddelow et al., 2017) by secreting proinflammatory cytokines and complement components (Lian et al., 2016). Moreover, antigen-presenting MG and MФ mediate T cell activation through MHC class II, and our transcriptomic data show an upregulation of C3, C1ra, and H2-Q10 transcripts in WT myeloid cells during EAE, raising the possibility that GRIP1 contributes to their expression.

Ongoing studies are investigating the CNS transcriptomic cell composition during homeostasis and disease in an effort to identify key molecular signatures driving neuroinflammation in MS (Butovsky et al., 2014; Hickman et al., 2013; Jordão et al., 2019). Our scRNAseq results illustrate the complexity of this task, given the plasticity of immune cell gene expression that is specified not only by inflammatory signals and cell–cell interactions but also by the CNS topographical subregions (Greenhalgh et al., 2020). Our results suggest that activated myeloid cells in EAE exist in several states with a gradient of gene expression between these states. Furthermore, the similarity of activated MФ and MG makes it difficult to distinguish these cells. Although the use of homeostatic MG markers as a proxy to determine the identity of scRNAseq subpopulations remains a possibility, the varying degree of MG activation leads to expression distribution rather than distinct clusters.

### GRIP1 and the type I IFN pathway in MS/EAE

GRIP1 is an established GR coregulator driving anti-inflammatory signaling of GCs in MФ and in vivo. We were surprised that its deletion showed no impact of the therapeutic efficacy of Dex in EAE in our system. We note, however, that the high Dex doses chosen matched those of GCs used to treat MS patients during flares. Hence, we cannot exclude the possibility that more thorough titration of Dex might uncover the phenotype of the GRIP1-cKO.

In contrast, therapeutic properties of IFN-β were clearly abolished in our GRIP1-cKO mice. Although this finding is internally consistent with well-documented neuroprotective effects of IFN-β in MS/EAE (Bermel and Rudick, 2007; Teige et al., 2003; Touil et al., 2006) and the role of GRIP1 in the type I IFN pathway (Flammer et al., 2010; Reily et al., 2006), a broader question regarding the type I IFN network in MS/EAE immunopathology remains unresolved. First, although efficacious in RRMS, IFN-β is ineffective at delaying secondary progressive MS, a more advanced disease type developed by most RRMS patients (Panitch et al., 2004; Zhang et al., 2015). Second, almost half of RRMS patients do not respond to IFN-β or develop resistance after several months of treatment (Bertolotto, 2004; Huber et al., 2015). Finally, in a subset of patients with a prominent Th17 response (as well as in Th17-induced EAE), IFN-β is proinflammatory and exacerbates pathology (Axtell et al., 2010; Axtell et al., 2011). Some studies linked this duality of IFN-β actions to whether it is produced in the CNS versus the periphery (Khorooshi et al., 2015; Reder and Feng, 2014). We observed an upregulation of IFN signature in the CNS of WT relative to GRIP1-cKO mice. Indeed, gene expression analysis of the whole brain and spinal cord and the purified spinal cord–derived myeloid cells showed an upregulation of key type I IFN pathway genes (*Irf1*,* Irf7*,* Irf4*,* Oas3*,* ifitm1*) in WT mice with more severe disease. This finding challenges the notion that IFN-β properties in MS/EAE are uniformly protective or that MG near CNS lesions during EAE upregulates endogenous IFN-β production as part of the healing process (Khorooshi et al., 2015; Kocur et al., 2015). Although not feasible at this stage, it could be informative to examine GRIP1 expression levels in patients with MS as related to disease severity or response to therapy.

We envision that the ability of GRIP1 to interact with different IRFs is linked to the complexity of IFN pathway functions in neuroinflammation and, indeed, to the two distinct phenotypes of GRIP1 deletion in our EAE model. Conceivably, GRIP1 binding to IRF3/7 promotes inflammatory signaling in myeloid cells, whereas downstream of IFNAR, the GRIP1–IRF9 interaction mediates IFN-β therapy. If so, GRIP1 deletion will attenuate IRF3/7-dependent myeloid cell–induced neuroinflammation but, at the same time, blunt the therapeutic effect of IFN-β. Currently, a subset of nuclear receptors and IRF proteins both represent viable candidates for enacting the unexpected proinflammatory function of GRIP1 in neuroinflammation.

Despite undeniable progress in dissecting immune cell pathways that mediate MS, there is no cure for this disease, and treatments mostly aim at alleviating symptoms. A dramatic recent improvement of transcriptomic tools yielded a better understanding of CNS-resident and infiltrating cell type diversity and their shifting activation states. This is particularly applicable to MG whose transcriptional states evolve as a function of localization, age, and disease (Prinz et al., 2019). Identifying GRIP1 as a novel player that specifies a subset of these transcription programs during neuroinflammation and IFN-β therapy should help elucidate potential therapeutic targets for MS management.

## Materials and methods

### Mice

C57BL/6 mice (National Cancer Institute, Charles River Laboratories) and their transgenic derivatives were maintained in the Hospital for Special Surgery (HSS) Animal Facility in full compliance with institutional guidelines approved by the HSS Animal Care and Use Committee. Homozygous WT (WT/WT;GRIP1^fl/fl^), LysM-WT (LysM-Cre;GRIP1^wt/wt^), and GRIP1-cKO (LysM-Cre;GRIP1^fl/fl^) mice (see Table S7 for key resource listing) were generated previously (Coppo et al., 2016). For all tissue and cell collection, mice were euthanized via CO_2_ inhalation.

### EAE induction and treatments

#### Active EAE

Mice 8 to 12 wk of age were immunized s.c. at two sites on the lower back with 100 µl (200 µl/mouse) of an emulsion containing 1:1 of MOG_35–55_ (200 µg/mouse; Rockefeller University Proteomics Resource Center) in PBS (Corning) + H37Ra *Mycobacterium tuberculosis* (1 mg/mouse) in CFA. Mice received PTX (200 ng/mouse; List Biological Laboratories) i.p. on days 0 and 2. EAE was evaluated daily by weighing the mice and scoring them following modified Hooke Laboratories mouse EAE scoring (each score is multiplied by 2); briefly, 0 = when picked up by base of tail, the tail has tension and is erect; 1 = tip of tail is limp; 2 = limp tail; 3 = limp tail and hind leg inhibition; 4 = limp tail and weakness of hind legs; 5 = limp tail and dragging of hind legs; 6 = limp tail and complete paralysis of hind legs; 7 = limp tail and complete paralysis of hind legs in addition to hind quarters are flat, giving the appearance of a hump on the front quarters of the mouse; 8 = limp tail, complete hind legs, and partial front leg paralysis; 9 = complete hind and front legs paralysis; 10 = moribund or dead. Wet food was supplied when the animals had paralysis of one or two hind legs, and mice were sacrificed if they reached a score of 8.0.

#### Passive EAE

WT female 8- to 12-wk-old donor mice were immunized as described for active EAE but did not receive PTX. At DPI10, spleen and dLNs (inguinal, axillary, and brachial) were collected and passed through a 70-µm filter (Falcon) to obtain a single-cell suspension. Cells were incubated with 1× RBC lysis buffer (BioLegend) for 5 min at 4°C. After centrifugation (500 ×*g* for 5 min at 4°C), cells were cultured in complete T cell media (5 × 10^6^ cells/ml), DMEM (Corning) supplemented with 50 µg/ml MOG_35–55_, 25 ng/ml IL-12, and 10 ng/ml IFN-γ (Thermo Fisher Scientific). After 72 h, CD4^+^ T cells were collected using the Dynabeads Untouched Mouse CD4 Cells Kit (Thermo Fisher Scientific) and injected into recipient mice (10^6^ cells/mouse i.p.). PTX was injected (200 ng/mouse i.p.) at days 0, 2, and 3 after CD4^+^ cell transfer. Clinical scoring and disease severity were assessed as above.

#### Dex and IFN-β treatment

After active EAE was induced, mice were injected i.p. daily with Dex (50 mg/mouse; Sigma), IFN-β (10^4^ U/mouse; R&D Systems), or PBS from the day symptoms started (score 2.0, usually approximately DPI10) for 10 consecutive days.

### Histopathology and image analysis

Mice were sacrificed and perfused through the heart with 25 ml of PBS, followed by 25 ml of 10% neutral buffered formalin solution (Sigma). Whole spines were collected and fixed overnight in formalin. Spinal cords were extracted from bone in 70% ethanol and transferred to the histology core. Tissues were paraffin embedded and sectioned, and slides were stained with H&E or LFB for myelin. Immunohistochemistry was performed by the Laboratory of Comparative Pathology at the Weill Cornell Medical College using anti-CD3 antibodies for T cells and anti-Iba1 for MG and MФ.

Quantitative image analysis of spinal cord H&E slides for inflammatory loci was performed using National Institutes of Health ImageJ software (Schneider et al., 2012). Every slide was divided into equally sized quadrants; the number of quadrants positive for inflammatory pockets was counted and expressed as a percentage of the total number of quadrants. The same strategy was used to count the number of CD3^+^ cells per slide. Myelin areas were quantified as the ratio of the myelinated plaque area to the total white matter area, both measured on each section series using ImageJ. Measurements for all three parameters (inflammatory pockets, CD3^+^ cell counts, and myelinated areas) were expressed as mean ± SD for each genotype.

All Iba1 antibody–stained images were processed using the R package EBImage (Pau et al., 2010) as follows: (1) extracted blue channel of the image; (2) multiplied image array by 1.55 × *m*/0.43137, where *m* is the mean of image intensity; (3) converted image into a negative image and filtered out pixels of intensity >0.72; (4) applied fillHull function; and (5) identified and masked individual objects on the image. Faithfulness of object detection was manually reviewed. Area, perimeter, maximum radius, minimum radius, mean ± SD of radius, center of mass on x and y axes, elliptical eccentricity, and object angle were recorded for analysis. Only objects bigger than 50 pixels and smaller than 3,000 pixels were kept for further classification. To filter out the noncellular compartment from objects identified, a training set, composed of 277 cells and 277 noncellular objects randomly selected from among 1,614 manually classified and randomly selected objects, was used to build a random tree forest classifier (mtry = 3, ntree = 1,500; Liaw and Wiener, 2002). The classifier was validated with the rest of the manually classified objects, and overall accuracy was 93.9%. This classifier was then used to screen out noncellular objects from all objects identified previously. Measurements were scaled by ranking each of them across all objects. MG activation status classification was achieved by cutting the tree yielded by agglomerative hierarchical clustering (complete-linkage method) into three clusters. Sums of cells in each cluster were recorded for each image. The morphology of randomly selected 20 cells from each cluster was assessed: round = activated MG; bushy = activating MG; and dendritic = homeostatic MG.

### Isolation of CNS-infiltrating cells

Mice were sacrificed and perfused through the heart with 25 ml of PBS using an 18-gauge needle. Spinal cords were flushed out of the spines by inserting a 5-ml syringe needle filled with 1× PBS, minced with dissection scissors, and incubated in 1× HBSS containing 4,000 U/ml collagenase D (Roche) and 10 ng/ml DNase I (Roche) for 30 min at 37°C. Enzymatic digestion was stopped by adding 0.5 M EDTA to a 12.5 mM final concentration, and tissue was pipetted up and down with a Pasteur pipette to release cells. Samples were then filtered through a 70-µm mesh and washed with PBS supplemented with 2% FBS (Atlanta Biologicals) to remove collagenase D, and then pellets were purified using a 30%/70% Percoll density gradient (Sigma) to separate immune cells from myelin. After collection, immune cells were washed twice with PBS, counted, and stained for flow cytometry (antibodies panel described below).

### Antibodies and FACS analysis

Cells were suspended in 1 ml of FACS buffer (PBS with 2% FBS). After Fc receptor blocking with antimouse CD16/32 (BioLegend), cells were incubated with a mix of antibodies specific to analysis at 1:200 dilution each for 20 min, and dead cell marker 7-aminoactinomycin D (5 µl/sample) or DAPI (1 µl/sample) was added right before acquisition on a BD FACSCanto II device. Data were analyzed using BD FACSDiva software. Cell counts were reported to initial cell number determined after Percoll density gradient purification.

The antibody panel for CNS-infiltrating cells was as follows: Alexa Fluor 488 anti-CD45, APC anti-CD3, PE anti-CD11b, PE–cyanine 7 anti-F4/80, and 7- aminoactinomycin D (BioLegend). The antibody panel for RNAseq was as follows: Alexa Fluor 488 anti-CD45, APC anti-CD3, PE anti-CD11b, and DAPI (BioLegend).

### Restimulation of cells in vitro

Mixed T cells and APCs from dLNs and spleens were prepared from mice at DPI7 or DPI20, and single-cell suspensions were made using a 70-µm cell strainer. For spleens, following RBC lysis, single-cell suspensions were counted, resuspended at 8 × 10^6^ cells/ml, and restimulated overnight with 50 µg/ml MOG_35–55_ peptide and 1 µg/ml each of anti-CD3 and anti-CD28. Brefeldin A (5 µg/ml; Sigma-Aldrich) was added for the last 4 h of culture, and cytokine production from T cells was evaluated using a cytometric bead array (CBA; BD Biosciences).

### Serum cytokine measurements

Mice were sacrificed when one or more mice reached a clinical score of 8 or higher. Blood was collected from the heart with 18-gauge needles, centrifuged immediately to separate serum from RBC, and flash frozen on dry ice. Cytokine levels were measured using the BD CBA Mouse Inflammation Kit for IL-6, IL-10, MCP-1, IFN-γ, TNF, and IL-12p70 and the Mouse Th1/Th2/Th17 Cytokine Kit to quantify IL-2/TNF/IFN-γ for Th1, IL-6/IL-4/IL-10 for Th2, and IL-17A for Th17. Briefly, serum samples were diluted following kit instructions and incubated with a mix of antibody-conjugated beads, followed by addition of PE detection reagent. Fluorescence levels were assessed with the FACSCanto II device, and cytokine levels were determined by comparing the fluorescence intensity of the PE reagent and reporting it to a control standard curve for each cytokine.

### Neonatal mouse MG isolation

P0 mice were decapitated; olfactory nerves were cut; and cerebellum, midbrain, and meninges were carefully removed under a microscope. Meninges-free brains were pooled, washed, and digested in 0.25% trypsin with 10 mg/ml DNase I (Roche) for 10 min at room temperature. Then, cells were homogenized gently with a 1,000-µl pipette to break up tissue without killing cells, filtered through a 100-µm cell strainer (BD Biosciences), and placed into DMEM with 10% FBS in the presence of 20% L-cell–conditioned media. 24 h thereafter, cells were washed and observed, with media changed every 3–4 d, until a large number of MG started to appear on top of the astrocyte monolayer. At days 14–20, cells were trypsinized, and MG was isolated by Cd11b microbead (Miltenyi Biotec) selection. MG were plated at a density of 50,000 cells/cm^2^ in DMEM with 10% FBS and 1% penicillin-streptomycin in flasks precoated with 0.01% poly-l-lysine (Sigma) for a minimum of 2 h. On the next day, they were treated as indicated in the figure legends and harvested for RNA isolation.

### Immunoblotting

8–12-wk-old mice were killed, and BMMФ were generated as described elsewhere (Chinenov et al., 2012). In brief, tibia and femur BM was flushed and cultured in 1 g/liter glucose-containing DMEM with 20% FBS supplemented with 20% L-cell–conditioned media for 5 d. Adherent cells were then scraped, plated at 2 × 10^7^ in 150-mm plates in DMEM with 20% FBS, and cultured overnight before harvest. Mixed glial cultures from P0 pups were prepared as described above, and the astrocyte monolayer was collected after MG purification by percussing the flask with a flat palmar surface. Dorsal root ganglia were isolated from E15 pups’ spinal cords as described previously (Sleigh et al., 2016). For T cell purification, spleens were collected and crushed, and single-cell suspensions were prepared using a 70-µm cell strainer. RBC lysis was performed, and CD4^+^ T cells were isolated using the Dynabeads Untouched Mouse CD4^+^ Cells Kit.

Whole-cell extracts were prepared using a standard procedure in radioimmunoprecipitation assay buffer (10 mM Tris-HCl, pH 8.0, 1 mM EDTA, 0.5 mM EGTA, 140 mM NaCl, 5% glycerol, 0.1% Na-deoxycholate, 0.1% SDS, 1% Triton X-100). Proteins were fractionated by SDS-PAGE and transferred to a polyvinylidene difluoride membrane using a transfer apparatus according to the manufacturer’s protocols (Bio-Rad Laboratories). Membranes were incubated in 5% nonfat milk in TBST (10 mM Tris, pH 8.0, 150 mM NaCl, 0.5% Tween 20) in the presence of commercial primary antibodies against GRIP1 (ab10491, 1:2,000, Abcam; and 611319, 1:500, BD Biosciences) and heat shock protein 90 (4874S, 1:2,000; Cell Signaling Technology) at 4°C overnight, washed in TBST (three times for 5 min each), and incubated with secondary antirabbit or antimouse HRP conjugate (W4011 and W4021, 1:10,000; Promega) at room temperature for 60 min. Blots were washed with TBST (three times for 5 min each) and developed with an enhanced chemiluminescence system (Amersham Biosciences) according to the manufacturer’s protocols.

### RNA preparation and real-time qPCR

Total RNA was isolated from cells with the RNeasy Plus Micro Kit (QIAGEN) or from homogenized whole brains and spinal cords of perfused mice using TRIzol (Thermo Fisher Scientific) extraction. RNA samples were subjected to random-primed cDNA synthesis, and gene expression was analyzed by qPCR with Maxima SYBR Green/ROX/2× Master Mix (Fermentas) on the StepOne Plus Real-Time PCR System (Applied Biosystems) using the comparative cycle threshold method. PCR primers are listed in Table S6.

### Transcriptomic analysis

#### Bulk RNAseq and pathway analysis

Mice were killed, and immune cells were collected from the CNS as described above, stained (antibodies panel described below) in PBS with 2% FBS, and sorted at the Weill Cornell Flow Cytometry Core. F4/80^+^CD11b^+^CD45^high^ and F4/80^+^CD11b^+^CD45^low^ sorted cells were collected in 350 µl of RLT buffer, and total RNA was isolated using the RNeasy Plus Micro Kit. The integrity of RNA and the quality of sequence-compatible libraries were evaluated with the BioAnalyzer 2100 system (Agilent). RNA was poly(A) enriched, and paired-end sequencing–compatible RNAseq libraries were prepared by the Weill Cornell Epigenomics Core Facility and sequenced (HiSeq 2500; 50-bp single-end protocol) at a depth of 22 million to 29 million mappable reads/sample. Read quality evaluation and adapter trimming were performed using fastp. All reads that passed initial quality filtering were mapped to the mouse genome (mm10), and reads in exons were counted against GENCODE release 27 annotation with the STAR aligner (Dobin et al., 2013). Batch correction to account for sex and day of the experiment was performed using the surrogate variable analysis (sva) ComBat function in R (Leek et al., 2020). Differential gene expression analysis was performed with edgeR using a likelihood ratio test. Genes with low expression levels (<3 counts per million in at least one group) were filtered from all downstream analyses. Genes with unadjusted P values <0.01 and log_2_ fold change >1 were considered differentially expressed. Downstream analyses were performed in R using a Shiny-driven visualization platform (RNAseq DRaMA) developed at the HSS David Z. Rosensweig Genomics Research Center.

Briefly, myeloid gene expression was modeled as a batch-corrected average of gene expression in MФ (CD45^high^; *n* = 2–4) and MG (CD45^low^; *n* = 3). Differentially expressed genes as defined above were used to perform QuSAGE pathway analysis using the MsigDB 6.2 C2 set (curated gene sets). All gene sets with unadjusted P values <0.01 were log fold change sorted and visualized using RNAseq DRaMA.

#### scRNAseq amplification and library preparation

Myeloid cells from mice with EAE were obtained as described in the Bulk RNAseq and pathway analysis section. F4/80^+^CD11b^+^CD45^+^ cells were sorted and retrieved in PBS, and the scRNAseq libraries were constructed using Chromium Single-Cell 3′ Reagent Kit version 3 according to the manufacturer’s workflow (10x Genomics). Briefly, single suspensions of FACS-sorted cells were encapsulated into emulsion droplets at a concentration of 500 cells/µl using the Chromium Controller (10x Genomics) for target output of ∼5,000 cells/sample. After reverse transcription and droplet dissociation, cDNA was purified with Dynabeads and amplified by PCR (13 cycles). For library construction, resulting cDNAs were fragmented, size selected (450 bp) with solid-phase reversible immobilization beads, and PCR amplified (14 cycles). The sequencing libraries were subjected to final cleanup using solid-phase reversible immobilization beads and evaluated on an Agilent Bioanalyzer. The generated scRNAseq libraries were sequenced by the Weill Cornell Genomics Core on the Illumina NovaSeq system using a 28-8-98 paired-end cycle.

#### scRNAseq data analysis

scRNAseq analysis was performed in R version 4.0.1 software (R Core Team, 2019). Quality control metrics were calculated with scater (McCarthy et al., 2017), and low-quality cells with mitochondrial reads exceeding replica median +1× median absolute deviation and <250 genes per cell were filtered out. Genes were filtered out if detected in <0.5% of all cells. Normalization was then performed using a cell pool deconvolution method (Lun et al., 2016). Doublet cells were predicted with scran’s doubletCells function, and cells with the doublet score in the upper decile were excluded from further analysis. Cell cycle phases were assigned using scran’s cyclone function. Highly variable genes were selected by modeling the relationships between the gene squared coefficient of variation relationship and mean expression values. Highly variable genes with false discovery rate <0.1 were used for data dimensionality reduction with principal component (PC) analysis; the top 18 PCs were selected using jackstraw (Chung, 2020).

Replica integration was performed using fastMNN implementation of a mutual nearest neighbors algorithm from the batchelor package (Haghverdi et al., 2018) using the top 18 PCs and the number of nearest neighbors, *k* = 20. The integrated PCs were projected into two-dimensional UMAP space (Mclnnes et al., 2018) for visualization. To further ascertain cell subpopulations, we performed community clustering using the Louvain algorithm (R::igraph) applied to the shared nearest neighbor graph build using the scran::buildSNNGraph function with the number of nearest neighbors, *k* = 75, in mnn-corrected PC analysis space (18 dimensions). Cluster characteristics are shown in Table S1. To get insight into the identity of the Louvain clusters, we searched for cluster-specific markers using the Wilcoxon rank-sum test (Korsunsky et al., 2019). The U statistics of the test are proportional to receiver operating characteristic analysis area under the curve. In pairwise comparison between a given cluster and all other clusters (one against all), area under the curve is interpreted as the probability of a cell from one cluster having higher expression of a given gene than cells from other clusters. An automated cell type assignment was performed with singleR using training sets derived from the Immunological Genome Project database (Aran et al., 2019). In addition, we used PanglaoDB to identify putative cell identity and/or activation state for each individual cluster. To identify cell type and cell activation state transitions, we performed trajectory analysis with slingshot (Street et al., 2018) in UMAP1–UMAP2 coordinates. Because the Louvain cluster 3 is the only cluster heavily enriched with cell proliferation markers, and because singleR assigns several types of stem cells to this cluster, we assigned cluster 3 as a trajectory starting cluster. We further excluded Louvain clusters 8 and 9 that have a well-defined cell identity (combined T cells and neutrophils). Trajectory-associated markers were determined by fitting a general additive model with a locally estimated scatterplot smoothing term for each gene to model the relationships between gene expression and pseudo-time.

Finally, we determined differentially expressed genes between WT and GRIP1-cKO mice, stratified by Louvain or singleR clusters (Tung et al., 2017), using a pseudo-bulk approach as implemented in the scran package (Franzén et al., 2019). Briefly, pseudo-bulk samples were created for each Louvain cluster by aggregating counts for all cells with the same combination of genotype and sample (cluster 3 was excluded due to a low cell number in this cluster in the WT). Differential expression analysis was performed with the edgeR quasi-likelihood framework using the pseudoBulkDGE function of the scran package that allows simultaneous differential expression analysis in multiple clusters (Table S5).

### Data deposition

RNAseq of MG from WT and cKO P0 mice and of myeloid cells from spinal cords of WT and KO mice with EAE is available in the Gene Expression Omnibus database (GSE141721).

### Online supplemental material

Fig. S1 includes additional data related to Fig. 1, showing the gene expression profile in WT and GRIP1-cKO P0 MG in response to LPS, LPS + Dex, or LPS + IFN-β. Fig. S2 includes additional data related to Fig. 1 and Fig. 2, showing that EAE attenuation in GRIP1-cKO mice is independent of Cre expression, the lack of apparent phenotype in GRIP1-cKO at homeostasis, and our FACS gating strategy. Fig. S3 includes additional data related to Fig. 3, showing gene expression analysis in whole spinal cords of WT and GRIP1-cKO mice at homeostasis and at EAE DPI20, and the Th2 cytokine production from T cells isolated from WT and GRIP1-cKO mice at DPI7 and DPI20 and restimulated in vitro. Fig. S4 includes additional scRNAseq analysis related to Fig. 6. Fig. S5 includes additional data related to Fig. 7 indicating that Dex treatment reverses EAE similarly in WT and GRIP1-cKO mice. Table S1, Table S2, Table S3, Table S4, and Table S5 include datasets related to Fig. 6 scRNAseq analysis. Table S6, related to Fig. 3 A, Fig. S1 B, and Fig. S3 A, shows sequences of PCR primers used in this study. Table S7 lists key resources used in this study.

## Supplementary Material

Table S1 lists cell count by Louvain or singleR clusters.

Table S2 lists cluster-specific markers in Louvain clusters.

Table S3 reports differential expression analysis along slingshot trajectories.

Table S4 shows that Gene Ontology proliferative categories are enriched among genes with area under the curve >0.7 from cluster 3.

Table S5 reports differential expression analysis between cKO and WT cells, stratified by Louvain clusters.

Table S6 lists RT-qPCR primer sequences.

Table S7 lists key resources used in this study.
