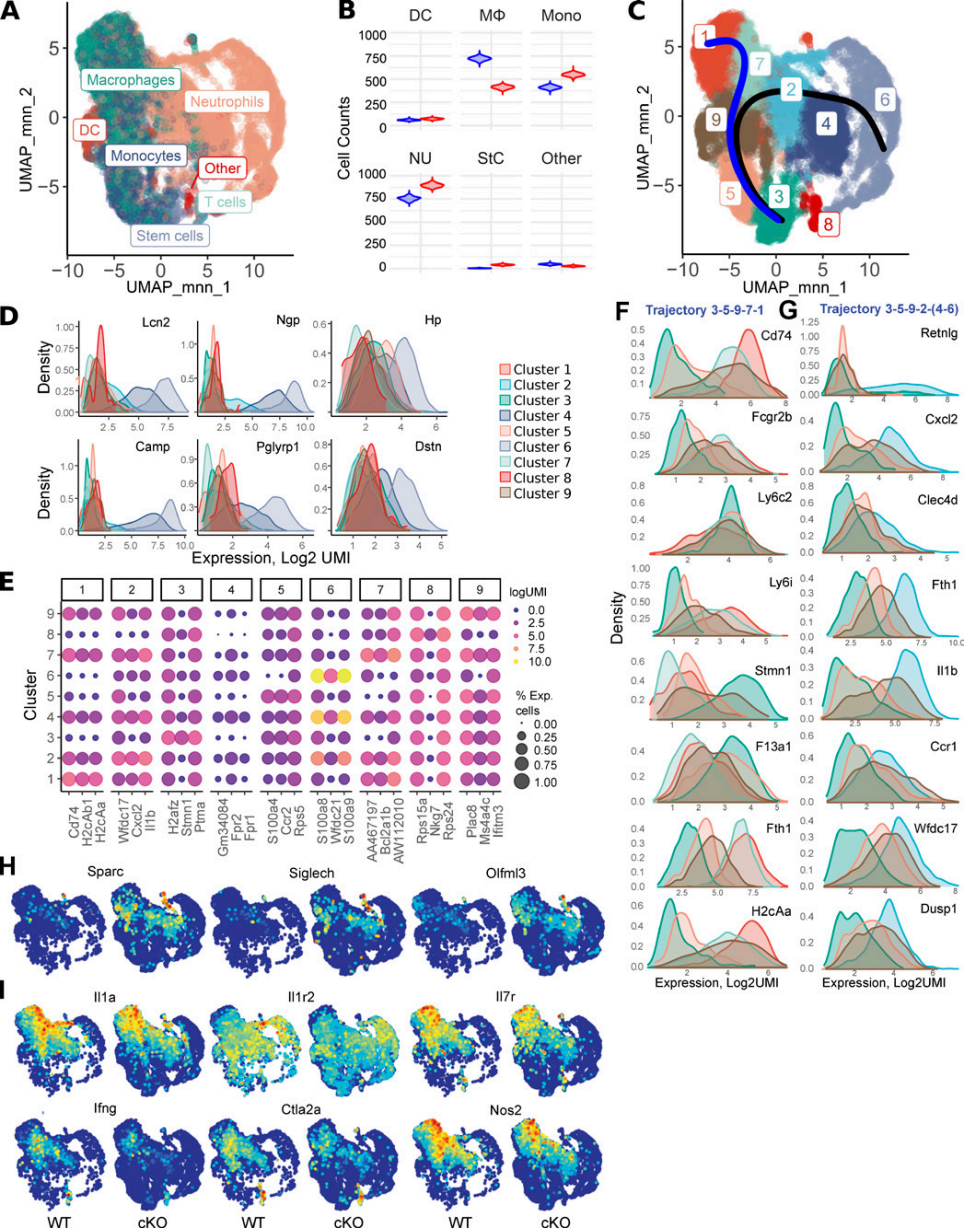# Correction: Transcription cofactor GRIP1 differentially affects myeloid cell–driven neuroinflammation and response to IFN-β therapy

**DOI:** 10.1084/jem.2019238611162020C

**Published:** 2020-11-23

**Authors:** Sanda Mimouna, David A. Rollins, Gayathri Shibu, Bowranigan Tharmalingam, Dinesh K. Deochand, Xi Chen, David Oliver, Yurii Chinenov, Inez Rogatsky

Vol. 218, No. 1 | 10.1084/jem.20192386 | October 12, 2020

*JEM* regrets that in the original version of this article, axes and cell type labels were missing in Fig. 6, A–C, as a result of a conversion error. The corrected figure is shown here. The errors appear only in PDFs downloaded before October 20, 2020.

**Figure:**