# Supplementary material for: Transcription cofactor GRIP1 differentially affects myeloid cell–driven neuroinflammation and response to IFN-β therapy
Source: J Exp Med. 2020 Oct 12;218(1):e20192386. doi: 10.1084/jem.20192386 (PMC7555412; doi:10.1084/jem.20192386)
Supplement: Table S7 — lists key resources used in this study. [file JEM_20192386_TableS7.docx]

### Table S7. Key resources table

###

| Reagent or resource | Source | Identifier |
| --- | --- | --- |
| Antibodies | | |
| Anti-mouse CD45 FITC (30-F11) | BioLegend | Cat #: 103122; RRID: AB_493531 |
| Anti-mouse/human CD11b PE (M1/70) | BioLegend | Cat #: 101208; RRID: AB_312791 |
| Anti-mouse CD3 APC (17A2) | BioLegend | Cat #: 100236; RRID: AB_2561456 |
| Anti-mouse F4/80 PE/Cy7 (BM8) | BioLegend | Cat #: 123114; RRID: AB_893478 |
| Purified anti-mouse CD3 (17A2) | BioLegend | Cat #: 100202; RRID:AB_312659 |
| Purified anti-mouse CD28 (37-51) | BioLegend | Cat #: 102102; RRID: AB_312867 |
| Purified anti-mouse CD16/32 (93) | BioLegend | Cat #: 101301; RRID: AB_312801 |
| Rabbit polyclonal anti-HSP90 primary antibody | Cell Signaling Technology | Cat #: 4874S; RRID: AB_2121214 |
| Rabbit polyclonal anti-KAT13 C/NCOA2 primary antibody | Abcam | Cat #: ab10491; RRID: AB_297230 |
| Anti-mouse IgG (H+L), HRP conjugate secondary antibody | Promega | Cat #: W4021; [AB_430834](http://antibodyregistry.org/AB_430834) |
| Anti-rabbit IgG (H+L), HRP conjugate secondary antibody | Promega | Cat #: W4011; [AB_430834](http://antibodyregistry.org/AB_430834) |
| Bacterial and virus strains | | |
| Difco *Mycobacterium Tuberculosis* H37 Ra | BD | Cat #: 231141 |
| Chemicals, peptides, and recombinant proteins | | |
| Viability Staining Solution 7-AAD | BioLegend | 420404 |
| DAPI | BioLegend | 422801 |
| IFN-γ recombinant mouse protein | Thermo Fisher Scientific | PMC4031 |
| IL-12 recombinant mouse protein | R&D | 419-ML-050 |
| Stock peptide MOG_35-55_  Amino Acid sequence : MEVGWYRSPFSRVVHLYRNGK | The Rockefeller University Proteomics Resource Center (28925) | RU105PROPQU184569 |
| Recombinant mouse IFNβ protein | Pbl Assay Science | 12401-1 |
| Pertussis Toxin from B. pertussis | List Biological Laboratories | #180  #181 |
| Collagenase D | Roche | 11088882001 |
| DNAse I | Roche | 10104159001 |
| Percoll | Sigma-Aldrich | P1644-500ML |
| TRIzol Reagent | Invitrogen | 15596026 |
| Brefeldin A | Sigma-Aldrich | CAS 20350-15-6 |
| Maxima SYBR Green/ROX qPCR | Thermo Fisher Scientific | K0223 |
| Critical commercial assays | | |
| Dynabeads Untouched Mouse CD4 Cells Kit | Thermo Fisher Scientific | 11415D |
| RNeasy Plus Micro Kit | QIAGEN | 74034 |
| Chromium single cell 3′ Reagent kits v3 | 10X Genomics | PN-1000092 |
| Mouse Inflammation Kit (RUO) | BD | 552364 |
| Mouse Th1/Th2/Th17 Cytokine Kit (RUO) | BD | 560485 |
| CD11b MicroBeads, human and mouse | Miltenyi Biotec | 130-049-601 |
| Mini Trans-Blot Cell | BIO-RAD | 1703930 |
| Pierce ECL Western Blotting Substrate | Thermo Fisher Scientific | 32209 |
| Deposited data | | |
| RNA-seq on P0 microglia | This paper | GEO submission GSE141721 |
| Bulk RNA-seq on myeloid cells from the CNS of WT and KO mice | This paper | GEO submission GSE141721 |
| Single cell RNA-seq on myeloid cells isolated from the CNS WT and KO mice | This paper | GEO submission GSE141721 |
| Experimental Models: Organisms/Strains | | |
| Mouse: LysM-Cre;GRIP1^wt/wt^ | Coppo et al., 2016 | N/A |
| Mouse: LysM-Cre;GRIP1^fl/fl^ | Coppo et al., 2016 | N/A |
| Mouse: wt/wt;GRIP1^fl/fl^ | Coppo et al., 2016 | N/A |
| Oligonucleotides | | |
| See Table S1 for list of quantitative RT-PCR primers | This paper | N/A |
| **Software and algorithms** | | |
| ImageJ | Schneider et al., 2012 | https://imagej.nih.gov/ij/ |
| BD FACSDiva v6.2 | BD Biosciences | N/A |
| FlowJo v10.4 | Tree Star | RRID: SCR_008520 |
| Prism v7 | GraphPad | RRID: SCR_002798 |
| *STA*R aligner | Dobin et al., 2013 | N/A |
| R | 4.0.1 | <https://cran.r-project.org/> |
| Matrix | 1.2.18 | <https://cran.r-project.org/> |
| magrittr | 1.5 | <https://cran.r-project.org/> |
| data.table | 1.13.0 | <https://cran.r-project.org/> |
| scater | 1.16.2 | Bioconductor |
| scran | 1.16.0 | Bioconductor |
| plotly | 4.9.2.1 | <https://cran.r-project.org/> |
| presto | 1.0.0 | <https://github.com/immunogenomics/presto> |
| slingshot | 1.6.1 | Bioconductor |
| gam | 1.2 | <https://cran.r-project.org/> |
| batchelor | 1.4 | Bioconductor |
| edgeR | 3.30.3 | Bioconductor |
| enrichR | 2.1 | <https://cran.r-project.org/> |
| igraph | 1.2.5 | <https://cran.r-project.org/> |
| jackstraw | 1.3 | <https://cran.r-project.org/> |
| SingleCellExperiment | 1.10.1 | Bioconductor |
| SummarizedExperiment | 1.18.2 | Bioconductor |
| rnaseqDRaMA | 1.4.1 | <https://gitlab.com/hssgenomics/Shiny> |
